# Enhanced Contour‐Deviant Mismatch Negativity and Mnemonic Representations in Older Musicians

**DOI:** 10.1111/ejn.70576

**Published:** 2026-06-16

**Authors:** Jennifer A. Bugos, Ricky Chow, Shimin Mo, R. Shayna Rosenbaum, Claude Alain

**Affiliations:** ^1^ School of Music, College of Design, Art & Performance University of South Florida Tampa Florida USA; ^2^ Department of Psychology and Centre for Vision Research York University Toronto Ontario Canada; ^3^ Rotman Research Institute Baycrest Academy for Research and Education Toronto Ontario Canada; ^4^ Department of Psychology University of Toronto Toronto Ontario Canada; ^5^ Institute of Medical Science University of Toronto Toronto Ontario Canada

**Keywords:** aging, electroencephalography, episodic memory, mismatch negativity, music, pattern separation

## Abstract

Instrumental music performance is associated with enhanced perceptual processing as evidenced by auditory discrimination and speech‐in‐noise perception. However, little is known about the extent to which auditory perceptual processes support cognition in aging. We investigated whether music training enhances perceptual precision in 26 older amateur and professional musicians (62–85 years, 16 females) and 25 older nonmusicians (61–82 years, 13 females). Participants completed a novel auditory mnemonic discrimination paradigm while electroencephalography (EEG) was recorded. The mismatch negativity (MMN), an event‐related potential of change detection, was measured during a passive auditory oddball paradigm with standard and deviant pure‐tone sequences differing in pitch contour. Participants subsequently completed an incidental memory test for oddball stimuli (i.e., targets) among similar lure sequences (matched for frequency but differing in contour) and dissimilar foil sequences (differing in frequency and contour), as well as a back‐to‐back perceptual discrimination task. Musicians showed enhanced amplitudes, left‐lateralized MMN source activity, and increased memory discriminability for targets relative to lures and foils, effects that were not explained by perceptual discrimination ability or MMN amplitude. No group differences were found in neural or behavioral measures on a mnemonic discrimination task involving everyday visual objects. Our results clarify the role of music training on precision in perception and auditory memory in older musicians compared with nonmusicians. Our findings underscore the contribution of musical engagement to perception and memory to the development of cognitive reserve in aging.

## Graphical abstract

Data showed enhanced MMN amplitudes in aging adult musicians compared with nonmusicians. Given that enhancements in MMN responses may be an indicator of preserved sensory processing in aging, music training may insulate age‐related decreases in sensory processing through preserved fidelity of encoding auditory information. Source localization showed left lateralization in musicians.

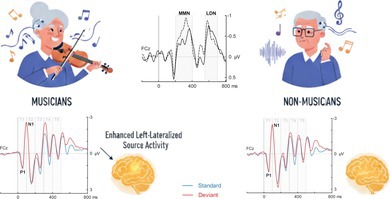

**Abbreviations**
A1: primary auditory cortex
AEP: auditory‐evoked potential
AMMT: Auditory MMN Memory Task
ANCOVA: analysis of covariance
BCa CI: bias‐corrected and accelerated confidence interval
BIC: Bayesian information criterion
CLARA: classical LORETA analysis recursively applied
CVLT‐3: California Verbal Learning Test, Third Edition
D‐KEFS: Delis–Kaplan Executive Function System
EEG: electroencephalography
ERP: event‐related potential
FA: false alarm
fMRI: functional magnetic resonance imaging
GAD‐7: Generalized Anxiety Disorder 7‐item scale
GLMM: generalized linear mixed‐effects model
GVIF^1/(2 × Df)^
: adjusted generalized variance inflation factor
HL: hearing level
IFG: inferior frontal gyrus
ISI: inter‐stimulus interval
LDI: lure discrimination index
LDN: late discriminative negativity
LMM: linear mixed‐effects model
LORETA: low‐resolution electromagnetic tomography
MET: Musical Ear Test
MMN: mismatch negativity
MoCA: Montreal Cognitive Assessment
MST: Mnemonic Similarity Task
PHQ‐8: Patient Health Questionnaire 8‐item scale
ROI: region of interest
SPL: sound pressure level
STG: superior temporal gyrus
TICS‐m: Modified Telephone Interview for Cognitive Status
WAIS: Wechsler Adult Intelligence Scale

## 1 Introduction

Musical performance requires integrating sensorimotor processing with continuous auditory feedback. Cross‐sectional studies show that musicians, compared with nonmusicians, demonstrate enhanced perceptual processing and executive functions (Alain et al. 2018; Strong and Mast 2019). For instance, musicians outperform nonmusicians on measures of central auditory processing such as figure‐ground perception in speech recognition tasks (Mankel and Bidelman 2018; Yoo and Bidelman 2019), gap detection tasks (Zendel and Alain 2012), and mistuned harmonic detection tasks (Zendel and Alain 2013). Studies also suggest benefits of musicianship on speech‐in‐noise processing (Parbery‐Clark et al. 2011; Zendel et al. 2019; Zendel and Alain 2012; but see Whiteford et al. 2025) and have shown correlations between musicianship duration and speech‐in‐noise performance (Zendel and Alain 2012). Notably, studies of speech perception found that neural activity in older musicians corresponds to perceptual functions and faster speech processing, as evidenced by early cortical responses (Bidelman and Alain 2015), speech categorization (Bidelman and Walker 2019), and perceptual learning paradigms (MacLean et al. 2024). Yet, little is known about the relationship between perceptual processing and episodic memory in older musicians. This study aimed to examine the role of extensive musical engagement on early sensory processing in older adults and determine the extent to which differences in perceptual abilities between older musicians and nonmusicians relate to changes in memory precision.

Research shows that music training can benefit perceptual and cognitive processing in older adults. Cross‐sectional music training studies comparing older musicians with extensive experience to age‐matched nonmusicians showed enhanced scores on measures of naming, executive functions, and auditory attention (Amer et al. 2013; Hanna‐Pladdy and MacKay 2011; Strong and Mast 2019). Longitudinal studies of group piano or percussion interventions for older adult novices revealed benefits to executive functions in cognitive flexibility (Bugos 2019; Mack et al. 2025), working memory (Degé and Kerkovius 2018), inhibitory control (Bugos 2010; Seinfeld et al. 2013), and verbal fluency (Bugos 2010; Bugos and Wang 2022). A possible reason for these enhancements is that musical engagement may strengthen sensitivity to structured auditory regularities and patterned sound sequences, thereby supporting the encoding and recognition of auditory information (Bonetti et al. 2025; Zak et al. 2026). The benefits of music training also seem to extend to the visual domain. For instance, musicians demonstrated enhanced selective attention compared with nonmusicians, which was not explained by increased sensorimotor integration (Rodrigues et al. 2013). Another study showed enhanced visuospatial working memory and inhibition in late middle‐aged adult musicians (Amer et al. 2013), suggesting far‐transfer effects from extensive musical engagement in a nonauditory domain. Even within the auditory domain, it is unclear whether the benefits of musical experience for sensory precision in aging extend into memory precision.

### 1.1 Perception and Memory Precision in Aging

Aging is often accompanied by declines in the specificity and precision of episodic memory (i.e., mnemonic discrimination; Korkki et al. 2020; Pishdadian et al. 2020; Stark et al. 2013; Yassa et al. 2011). One proposed mechanism for this decline is impaired pattern separation—the neural process that enables the encoding of distinct representations for similar or overlapping events (Rolls 2013, 2016). For instance, studies using the mnemonic similarity task (MST; Stark et al. 2013), a task that places demands on pattern separation, have shown age‐related deficits in distinguishing between previously studied everyday objects (targets) and highly similar but new items (lures), with intact performance for clearly dissimilar, unstudied items (foils) (Bowman et al. 2019; Huffman and Stark 2017; Pishdadian et al. 2020; Stark et al. 2013, 2015; Stark and Stark 2017; Toner et al. 2009; Yassa et al. 2011). Age‐related memory impairment may reflect deficits in encoding (i.e., sensory acquisition) and retrieval (Craik and Rose 2012; Langnes et al. 2019). Research reveals age‐related differences across sensory modalities in the encoding of simple stimuli (Alain et al. 2022; Reuter et al. 2013). These findings suggest that perceptual declines could contribute to memory impairments, with difficulties in perceptual discrimination at encoding leading to less distinct memory representations (Clinard et al. 2010; Gellersen et al. 2021; Roberts and Allen 2016). Yet, little is known about memory precision or the role of sensorimotor integration experience gained through music training on encoding in aging.

### 1.2 Sensory Processing and the MMN

Event‐related potentials (ERPs) provide a sensitive and temporally precise method for examining the neural mechanisms underlying sensory processing. Research using functional magnetic resonance imaging (fMRI) showed neural activity in the visual cortex during encoding predicted memory precision in young adults (Wing et al. 2020) and older adults (Bowman et al. 2019). In addition, activity of the auditory cortex has also been found to relate to prediction errors from violations of learned auditory sequences in associative learning paradigms (Nazimek et al. 2013). However, fMRI is limited in temporal resolution when compared with ERPs. Thus, ERP data are commonly acquired to measure the encoding of sensory information in a more process‐pure manner.

A well‐established neural correlate of change detection in perception is the mismatch negativity (MMN; Näätänen et al. 2007), an auditory ERP component that reflects automatic detection of deviations from expected sensory input or a bottom‐up neural index of sensory prediction errors (see Garrido, Kilner, Stephan, and Friston 2009 for a review). The MMN is typically elicited in oddball paradigms where a repetitive, standard stimulus (e.g., a pure tone) is occasionally replaced by a deviant stimulus differing in a perceptual feature (e.g., frequency, intensity, or duration). The repeated standard generates a prediction, whereas the deviant violates this prediction, triggering a prediction error response (Barron et al. 2020; Garrido, Kilner, Stephan, and Friston 2009). The MMN is the difference waveform between ERPs to deviant and standard stimuli, typically manifesting as a fronto‐central negative deflection peaking between 100 and 250 ms poststimulus.

The MMN component is particularly valuable for probing early sensory encoding and auditory sensory memory independent of attentional confounds. The amplitude and latency of the MMN are linked to perceptual discrimination processes and differ between musicians and nonmusicians: Larger amplitudes and shorter latencies are typically observed when the physical difference between standard and deviant stimuli is more salient, and earlier MMN onsets predict faster behavioral responses to changes in the auditory environment (Näätänen and Alho 1997; Schröger et al. 1992; Tiitinen et al. 1994). Several studies show that the MMN can index incidental learning of implicit abstract, hierarchical regularities based on a memory trace of the standard stimulus (see Paavilainen 2013 for review). Computational modeling by Teichert et al. (2025) showed that the MMN can index deviance detection based on information that persists on timescales beyond auditory sensory memory. Recent work showed that age attenuations in MMN amplitudes can partially explain age declines in subsequent episodic memory for oddball stimuli (Chow et al. 2025). Thus, the MMN may reflect stimulus‐specific adaptation to repetitive standard sounds and prediction error responses to violations of established auditory regularities (i.e., deviance detection; see Baldeweg 2007 and Wacongne et al. 2012). These findings highlight the MMN as a versatile marker of both basic, low‐level, and more complex abstract prediction mechanisms in auditory processing.

### 1.3 Musicians and MMN

Research suggests differences between musicians and nonmusicians in incidental learning through novel manipulations of musical elements such as pitch, melody, and dynamics. When compared with nonmusicians, musicians typically demonstrate increased MMN amplitudes, including violations to higher‐order, abstract regularities of auditory representations for tonal patterns (Alain et al. 1999; Fujioka et al. 2004; Horváth and Winkler 2004; Macdonald and Campbell 2011; Tervaniemi et al. 2006; Tervaniemi 2026). Tonal patterns in experiments with up to five pitches, regardless of key presentation, presented between 400 and 1000 ms in length, elicited an MMN in adults (Alain et al. 1999; Habermeyer et al. 2009; Tervaniemi et al. 2006). Research examining the sensory memory trace for tones in musicians and nonmusicians found a strong musician advantage for detecting deviants, which remained enhanced despite pattern length (Boh et al. 2011; Herholz et al. 2009). In addition, research in persons without music training suggests that the brain may automatically model pitch relationships based upon musical scale structures (Brattico et al. 2006). However, limited research has examined the MMN in older musicians, with only one study to our knowledge showing earlier MMN latencies to harmonic tone pitch deviants in a relatively small sample of older musicians than nonmusicians (55–70 years), though no difference in MMN amplitude was found (O'Brien et al. 2015). In another study, the MMN of older adult choral members showed more robust encoding of pitch and location features when compared with older nonmusicians (Pentikäinen et al. 2022). Although evidence indicates enhanced neural representations of auditory stimuli in musicians, it is unclear how these advantages are preserved in older adults.

Therefore, the present study examines the extent to which older musicians show enhanced indices of precision in auditory perception (as demonstrated by the MMN) and mnemonic discrimination in both auditory and visual domains. A passive oddball paradigm was used to elicit a contour‐deviant MMN. We hypothesized that musicians, compared with nonmusicians, would demonstrate enhanced encoding and memory performance for perceptually similar sound objects. To examine whether group differences in the contour‐deviant MMN predicted memory precision, subsequent memory for oddball stimuli was tested against similar lures and dissimilar foils. Compared with their nonmusician peers, we predicted that (1) older musicians would demonstrate enhanced MMN amplitudes to pitch contour deviants (i.e., temporal arrangement of pitched tones within sequence) and (2) enhanced subsequent memory for oddball stimuli. To examine the extent to which perceptual advantages from musicianship extend to memory precision in the visual domain, neural and behavioral measures of mnemonic discrimination for everyday objects were also tested between groups using the MST. Although the MST operates on a different premise with familiar and unfamiliar pictures for stimuli as compared with abstract, pure tone sequences in the auditory oddball task, the MST provides a useful measure of precision in visual memory. We also predicted higher lure discrimination on the visual MST compared with older adult nonmusicians.

## 2 Materials and Methods

### 2.1 Participants

Older musicians were recruited from community outreach (e.g., online advertisements, musician network mailing lists, and talks) in the Greater Toronto Area. Nonmusicians were recruited from the Rotman Research Institute participant database. Participants were classified as musicians if they reported at least five cumulative years of formal music training (operationalized as individualized private lessons on an instrument beyond general academic curricula or extracurriculars, such as secondary school music classes) and were concurrently playing or performing on an instrument at the time of testing. Participants were classified as nonmusicians if they reported fewer than four cumulative years of formal music training and were not concurrently learning or playing any musical instrument at the time of testing. Data from five older adults who did not fit the above criteria (e.g., reported between 2–5 years of formal music training and reported concurrently actively practicing or learning a musical instrument at the time of testing) were excluded from the study. All participants were right‐handed, reported fluency in English, and had no history of neurological conditions (e.g., stroke, transient ischemic attack, and traumatic brain injury) or formal diagnoses of a mood disorder, substance use disorder, or learning disabilities. Participants were excluded if they reported a history of chemotherapy or radiation therapy to the head or neck, or if they were concurrently taking medications known to substantially impact cognitive functioning (e.g., antidepressants or antipsychotics). Additionally, participants were excluded if they reported regular use of hearing aids or self‐reported hearing difficulties. No participant underwent a neuropsychological evaluation within the six months prior to testing. No participant scored below the cut‐off on the modified version of the Telephone Interview for Cognitive Status (TICS‐m). A pure‐tone audiogram of octave frequencies from 250 to 8000 Hz was administered to ensure age‐appropriate hearing thresholds. Data from one older nonmusician were excluded due to moderate hearing loss, operationalized as average thresholds exceeding an average of 35‐dB hearing level (HL) in both ears; therefore, data from this participant were used in analyses of visual tasks only. Data from one musician and one nonmusician were excluded due to cognitive status based on neuropsychological assessment (see below). All participants reported normal or correct‐to‐normal visual acuity.

The final sample of participants comprised 26 older musicians (62–85 years, 16 females) and 25 older nonmusicians (61–82 years, 13 females). A chi‐square test showed no significant difference in sex distribution across groups, *χ* ^2^(1, *N* = 51) = 0.47, *p* = 0.492. No group difference was found for years of education, *t*(49) = 1.62, *p* = 0.111, *d* = 0.455, *B*_10_ = 0.82; the sample was highly educated, with on average 17.73 years (SD = 2.51) of education for the musician group and on average 16.60 years (SD = 2.47) of education for the nonmusician group. All participants provided written informed consent and received monetary compensation for participation. The experimental protocol was approved by the Research Ethics Board at the Rotman Research Institute at Baycrest Centre and York University.

Musicians reported practicing a variety of instruments (i.e., piano, clarinet, flute, oboe, French horn, saxophone, and violin). All musicians reported training in the classical style and proficiency in reading standard staff notation. The musician sample reported practicing or performing an average of 9.13 cumulative hours (SD = 3.96 h) per week on an instrument. Eleven of the 26 older musicians reported proficiency on two instruments, and 10 reported proficiency on three or more instruments. Eighteen reported completing a professional degree in music performance (i.e., Bachelor's degree or higher). Twenty‐three musicians started music lessons on at least one instrument before the age of 18, and the other three musicians began their training between 30 and 50 years of age. Nineteen musicians reported playing any single instrument for 10 or more years. Thirteen reported not having taken formal music lessons for 15 years or more. At the time of testing, nine reported concurrently engaging in formal music lessons, 25 reported playing regularly in an ensemble, and three musicians reported engaging in music composition. One musician reported absolute pitch ability (i.e., the ability to label the pitch of a note without an external reference). Eight of the 25 nonmusicians reported having received private music lessons in their lifetimes; this subset reported, on average, 2.00 years of consistent formal music education (SD = 1.31). Only two nonmusicians received four years of formal music education in their adolescence and reported no formal music education in the past 50 years.

### 2.2 Neuropsychological Assessment

All older adult participants were administered the Montreal Cognitive Assessment (MoCA; Nasreddine et al. 2005) as a brief test of global cognitive function. Data from one musician and one nonmusician were excluded, given a score below a revised, less conservative cut‐off (i.e., below 23; Thomann et al. 2020). The Vocabulary and Matrix Reasoning subtests from the Wechsler Abbreviated Scale of Intelligence (Wechsler 2008) were administered to estimate crystallized and fluid intelligence, respectively. The Digit Symbol Coding subtest from the Wechsler Adult Intelligence Scale, 3rd Edition (WAIS‐III) was administered to measure processing speed. The Trail‐Making Test (Forms A and B) assessed psychomotor speed and cognitive flexibility (Reitan and Wolfson 1993). Episodic memory performance was assessed using the 3rd edition of the California Verbal Learning Test (CVLT‐3; Delis et al. 2017), along with the Incidental and Free Recall trials of the WAIS‐III Digit Symbol Coding Test. The WAIS‐IV Digit Span subtest (Wechsler 2008) was administered to assess auditory attention and working memory span. The Verbal Fluency subtest from the Delis–Kaplan Executive Function System (D‐KEFS; Delis et al. 2012) was administered, including trials for measuring phonemic fluency, semantic fluency, and cognitive flexibility. The Musical Ear Test (Wallentin et al. 2010) was administered to test musical aptitude in melodic and rhythmic domains. To assess depressive and anxious symptoms, all participants completed the Generalized Anxiety Disorder 7‐item scale (GAD‐7; Spitzer et al. 2006) and the Patient Health Questionnaire 8‐item scale (PHQ‐8; Kroenke et al. 2009).

### 2.3 Procedure and Electroencephalography (EEG) Acquisition

All participants completed two computerized tasks that examined mnemonic discrimination, the ability to precisely differentiate similar events or objects in episode memory. Participants first completed the MST, a well‐established measure of mnemonic discrimination that uses everyday visual objects. This task is thought to place demands on pattern separation by testing episodic memory for old target objects against perceptually similar lures and dissimilar foils (Stark et al. 2013). Subsequently, participants completed a novel auditory memory task—the Auditory MMN Memory Task (AMMT)—which evaluates mnemonic discrimination in the auditory domain using unfamiliar tone sequences lacking musicality (i.e., constructed irrespective of musical scale). This task, previously called the Auditory Pattern Separation Task (Herman et al. 2023), was administered following the MST. Participants were offered an optional break between the two tasks. To reduce fatigue, adults (aged 60+) completed the memory tasks on a separate day from neuropsychological assessments. All participants completed both sessions within two months, and no participant withdrew from the study. All participants completed the MST and the AMMT while seated in a recliner in a double‐walled, sound‐attenuated booth.

EEG data were recorded using 66 Ag/AgCl scalp electrodes with a BioSemi ActiveTwo system (BioSemi V.O.F., Amsterdam, Netherlands), following the 10–20 international electrode placement system. A common‐mode sense and driven‐right‐leg electrode served as references. Ten additional electrodes were placed below the hairline to monitor ocular activity and enhance coverage, including both mastoids, preauricular points, lateral points neighboring the outer canthi of the eyes, inferior to each eye, and two frontotemporal locations. EEG data were continuously digitized at 512 Hz (DC–100‐Hz bandwidth) and stored offline for analysis. EEG data for the MST are reported in Table S1.

### 2.4 AMMT

The AMMT comprised three stages: the oddball phase (i.e., incidental encoding phase), a surprise recognition test, and a same‐different perceptual discrimination task. Six distinct tone sequences were created, including two targets (standard and deviant), three lures, and one foil. Each sequence consisted of five 100‐ms pure tones, with tones arranged in different temporal orders to manipulate pitch contour. During the oddball phase, only two of these sequences (i.e., a standard and a deviant) were used, with all six sequences used in the latter two stages. Only the temporal arrangement of pitch order was changed between each tone sequence, and no rhythmic changes were introduced. Stimuli were created using Audacity (version 2.3.3) and incorporated 10‐ms onset and offset ramps to avoid auditory clipping. Target and lure sequences were designed to be highly similar in pitch contour while preserving the same set of pitches (i.e., comparable mean frequency). The foil sequence used the identical pitches transposed down one octave, making it perceptually distinct from the other sequences. Pitch contours were created to avoid recognizable Western musical scales or melodies, yielding abstract, amelodic sequences without clear tonal structure and thereby minimizing the influence of prior musical experience on memory. Auditory stimuli were presented at 85‐dB SPL through Etymotic ER‐3A insert earphones.

#### 2.4.1 Oddball Phase

Participants were presented with 1000 auditory sequences (700 presentations of the standard, 300 presentations of the deviant) in randomized order over a 25‐min session while watching a muted movie. This ratio of standard to deviant presentation minimized the inherent imbalance between standards and deviants in an oddball paradigm, while ensuring sufficient deviance for MMN generation as shown from our prior work (Chow et al. 2025; Herman et al. 2023). They were instructed to attend the movie, and compliance was monitored via webcam. Each sequence lasted 500 ms, followed by a jittered ISI between 900 and 1150 ms (in 50‐ms steps). Standard and deviant sequences differed in pitch contour (while matched in average frequency across tones) to elicit the MMN.

#### 2.4.2 Recognition Test Phase

After encoding, participants completed a surprise recognition test for the tone sequences presented during the oddball phase. Test probes included the original standard and deviant sequences (targets), three novel lure sequences with pitch contours similar to targets, and one foil sequence differing in contour and average frequency. To ensure task performance placed demands on long‐term episodic memory rather than on short‐term and working memory (Friedman and Johnson 2000; Stark et al. 2015), the test phase began after a 2‐ to 3‐min break during which instructions were provided. Participants identified each sequence as “old” or “new” in the context of the encoding phase using a keyboard button press. Each of the six sequence types (two targets, three lures, one foil) was presented 10 times in randomized order, totaling 60 trials (20 targets, 30 lures, 10 foils). The task was self‐paced, with a 500‐ms delay between each sequence and the response prompt.

##### 2.4.2.1 Perceptual Discrimination Task

In the final phase, participants completed a same‐different perceptual discrimination task to ensure they could distinguish among the sequences presented earlier. Although not central to the current study, this phase assessed perceptual discrimination abilities in the auditory domain. Each trial involved two sequences played back‐to‐back with a 500‐ms ISI. Participants then judged whether each pair was identical or different. Each tone sequence was paired both with itself (forming identical pairs) and with all other sequences. The task comprised three conditions: identical, similar, and dissimilar pairs. Similar pairs consisted of all possible nonidentical pairings of target and lure sequences (e.g., Lures 1 and 2), whereas dissimilar pairs included any nonidentical combinations involving the foil sequence with the remaining sequence types (e.g., Foil and Lure 1). All nonidentical pairs were presented in both possible orders. Within the identical condition, each of the six tone sequences was paired with itself and presented four times throughout the task, yielding 24 trials. Each unique nonidentical pairing was presented twice, yielding 40 similar and 20 dissimilar trials (resulting in a total of 84 trials across the task). This task was self‐paced and used a forced‐choice format, with responses recorded via keyboard button press.

### 2.5 MST

All participants completed the MST as a visual mnemonic discrimination task that placed demands on pattern separation for everyday objects. It included two phases: an incidental encoding phase and a recognition test. During encoding, participants viewed 128 images of everyday objects and categorized each as “indoor” or “outdoor.” In the subsequent test phase, participants were presented with 192 images and identified each as “old,” “similar,” or “new” relative to the encoding phase. These images comprised previously viewed targets, perceptually similar lures, and new foils. Each image was displayed for 2 s, with a 500‐ms inter‐stimulus interval (ISI). Participants were randomly assigned to one of two equivalent image sets (Set C or Set D). Responses were made via button press on a computer keyboard. EEG was recorded for both phases; results of analyses from only the test phase are reported in the present paper.

### 2.6 Data Preparation

#### 2.6.1 EEG Preprocessing

Preprocessing of ERPs was conducted using BESA Research software (version 7.1; MEGIS GmbH, Gräfelfing, Germany). Continuous EEG recordings were re‐referenced to the average reference and filtered with a 0.53‐Hz high‐pass filter (forward, 6 dB/octave) and a 40‐Hz low‐pass filter (zero‐phase, 24 dB/octave). Electrodes demonstrating substantial artefacts or drift (e.g., due to head or body movements) were interpolated using spherical spline interpolation (Picton et al. 2000); no more than 10% of channels were interpolated per participant. To correct for ocular artefacts (such as eye blinks and horizontal eye movements), a spatial filtering approach was used based on Berg and Scherg (1994) and Ille et al. (2002); spatial topographies that best accounted for blinks and vertical/lateral eye movements for each participant were marked and then subtracted from the continuous EEG recording.

##### 2.6.1.1 AMMT Oddball

For each participant, EEG data from the passive oddball paradigm were segmented into 1000‐ms epochs with a 200‐ms prestimulus baseline. These epochs were additionally screened for artefacts, and those with peak‐to‐peak fluctuations exceeding ±60 μV per channel were excluded from further analysis. For musicians, this excluded on average 9.45% (SD = 7.95%) of standard trials and 7.48% (SD = 6.91%) of deviant trials per participant. For nonmusicians, this excluded on average 9.06% (SD = 8.14%) of standard trials and 7.17% (SD = 6.25%) of deviant trials per participant; this did not differ between groups, *F*(1, 49) = 0.89, *p* = 0.351, *η* ^2^ *G* = 0.018. After artefact rejection, the remaining epochs were averaged separately for the standard and deviant conditions and baseline‐corrected using the 200‐ms prestimulus interval. To visualize the MMN component, the difference waveform was computed for each participant by subtracting the averaged standard waveform from the deviant waveform. Based on prior investigations using the same paradigm (Chow et al. 2025, 2026; Herman et al. 2023), the late discriminative negativity (LDN) component was also expected to be observed as a late deviance‐related potential that indexed higher‐order, auditory gestalt representations based on higher‐order abstract violations to pitch contour.

To investigate group differences in neural generators underlying the MMN, source activity was modeled using an iterative application of low‐resolution electromagnetic tomography (LORETA), termed classical LORETA analysis recursively applied (CLARA). This imaging approach provides focal localizations of the source activity by weighting images with a reduced source space in each iteration. Two iterations were performed; in the second, the image was spatially smoothed with a half‐width of one voxel. The regularization parameters were set to a single‐value decomposition cutoff at 0.01%, and voxels with amplitudes in the lowest 10% of the first image were set to zero to reduce the effective source image. This thresholded image was then used to define a voxel‐wise spatial weighting term for the next iteration, and a new LORETA image was computed incorporating this weighting. The voxel size in Talairach space was set to 7 mm. A four‐shell ellipsoidal head model with a head radius of 85 mm, and thicknesses for scalp, skull, and cerebrospinal fluid were set at 6, 7, and 1 mm, respectively. The relative conductivities were 0.33, 0.33, 0.0042, and 1 S/m for brain, scalp, skull, and cerebrospinal fluid, respectively. To ensure source images represented deviance‐related activity from oddball detection, CLARA images were computed on difference waveforms generated from the subtraction between standard and deviant trials (rather than standard and deviant waveforms separately). Source reconstructions were then compared between groups using a two‐tailed independent‐samples permutation *t*‐test.

##### 2.6.1.2 MST Test Phase

EEG data from the test phase were segmented into 1500‐ms epochs for each participant, including a 200‐ms prestimulus baseline. The epochs were then averaged according to condition (i.e., Target, Lure, and Foil); only epochs containing correct responses (i.e., “old” for Target, “similar” for Lure, and “new” for Foil). Epochs were also averaged to a fourth condition for lure false alarms (FA Lure condition, i.e., lures incorrectly classified as “old”) to examine false recognition of lures. To ensure adequate number of trials per condition, a closer inspection of trial counts revealed inadequate trial accounts for the correct Lure condition (on average 18.40 trials [SD = 7.66] for musicians, on average 17.00 trials [SD = 9.85] for nonmusicians, with only 30 participants with 15 or more trials with correct responses to lures) and were deemed not suitable for analysis due to low statistical power. Epochs were then screened for additional artefacts, and those exceeding ±120‐μV peak‐to‐peak amplitude were excluded from further analysis. The percentage of trials excluded did not differ significantly across groups (*F*(1, 48) = 0.105, *p* = 0.748, *η* ^2^ *G* = 0.002) (for musicians: 8.89% [SD = 8.50%] for Target, 9.04% [SD = 10.07%] for Foil, and 9.40% [SD = 8.37%] for the FA Lure condition; for nonmusicians: 8.09% [SD = 8.76%] for Targets, 9.22 [SD = 8.93%] for Foil, and 7.85% [SD = 7.60%] for the FA Lure condition). The remaining conditions were comprised of moderate trial counts (for musicians: on average 48.04 trials [SD = 6.95] for Target, 44.64 [SD = 8.45] for Foil, and 31.6 [SD = 6.42] for the FA Lure condition; for nonmusicians: on average 46.48 trials [SD = 9.13] for Target, 42.00 [SD = 10.73] for Foil, and 31.96 [SD = 8.53] for the FA Lure condition), which did not differ between groups, *F*(1, 48) = 1.51, *p* = 0.225, *η* ^2^ *G* = 0.014. Epochs were then baseline‐corrected using the 200‐ms prestimulus interval.

From these data, the FN400 (as an index of familiarity; Curran 2000) was expected to be visible as a frontocentral modulation between waveforms from correctly identified targets compared with correctly rejected foils, with a greater negativity for foils than targets. Similarly, the FN400 was also expected to be visible between waveforms from falsely recognized lures compared with correctly rejected foils. A greater FN400 in this contrast would signify greater false recognition of lures and worse mnemonic discrimination. Therefore, two difference waveforms were computed for each participant. To examine group differences in the old–new effect in episodic memory, the old–new difference waveform was created by subtracting the averaged Foil from Target conditions. Another difference waveform was created by subtracting the Foil condition from the FA Lure condition to examine group differences in false lure recognition. Given only moderate trial counts for the condition and to reduce the variability of individual participant waveforms, these difference waveforms were additionally filtered using a 20‐Hz low‐pass filter (zero‐phase, 24 dB/octave).

### 2.7 Behavioral Measures

#### 2.7.1 AMMT

Sensitivity metrics (i.e., *d*‐primes and response bias) were computed for the test phase: target–foil discriminability as a measure of the conventional old–new recognition performance in episodic memory, defined as *z* (old|targets)–*z* (old|foils), and target–lure discriminability as a measure of mnemonic discrimination, calculated as *z* (old|targets)–*z* (old|lures). As a measure of response bias, the criterion *c* was calculated per participant for each of target–lure and target–foil discriminability. Each measure was scaled by its corresponding number of trials (20 target trials, 30 lure trials, and 10 foil trials). This yielded a criterion c value for each of the two contrasts that reflected the tendency to respond “old” regardless of stimulus type, with positive values indicating a conservative bias and negative values indicating a liberal bias. Both *d*‐prime and *c* measures were based on loglinear‐adjusted *d*‐prime values, correcting for extreme response rates using the method outlined by Hautus (1995) using the *psycho* R package (Makowski 2018). Although standard tone sequences occurred more frequently than deviant ones during encoding (at a seven to three ratio), Wilcoxon signed‐rank tests indicated no significant differences in hit rates between the two target types across the sample (*W* = 486.0, *z* = 1.34, *p* = 0.180, *r*_*rb*_ = 0.25) and no significant interaction between group and target subtype (*F* = 1.27, *p* = 0.265, *η* ^2^ *G* = 0.009). Data from one musician was excluded from this task due to near‐ceiling response bias for “new” across the task.

For the perceptual discrimination task, hit rates were computed for three conditions: correctly identifying identical pairs as *same*, identifying similar pairs (i.e., target–lure or nonidentical lure pairs) as *different*, and identifying dissimilar pairs (i.e., foil sequence paired with either target or lure sequence) as *different*. Data from one nonmusician were excluded from this task due to not understanding the instructions.

##### 2.7.1.1 MST

Data from one nonmusician were excluded due to color‐blindness that was self‐reported after study participation, and data from another nonmusician were excluded due to falling asleep during the majority of the test phase; data were included from one nonmusician who did not participate in the AMMT due to mild hearing loss (total of *n* = 24 nonmusicians). Trials from the test phase were excluded from analysis if responses occurred outside the 2500‐ms response window. To control for accidental button presses, responses that occurred faster than 200 ms were excluded from accuracy calculations. On average, 1.18% (SD = 1.62%) of trials were excluded per participant in the musician group, and 3.80% (SD = 4.62%) for the nonmusician group, with a greater proportion of trials trimmed for nonmusicians (*W* = 163.5, *z* = 2.92, *p* = 0.004, *r*_*rb*_ = 0.476). Calculation of hit rates was performed for each of the three response categories, i.e., correctly identifying targets as “old,” lures as “similar,” and foils as “new.” Old–new recognition performance was computed by subtracting the false‐alarm rate to foils from the hit rate to targets (i.e., old|target–old|foil). Mnemonic discrimination was calculated as the difference between the proportion of lures correctly identified as “similar” and the proportion of foils incorrectly identified as “similar” (i.e., similar|lure–similar|foil).

### 2.8 Data Analysis

#### 2.8.1 ERPs

ERPs were analyzed using a data‐driven, cluster‐based permutation test, identifying statistically significant spatiotemporal clusters reflecting amplitude differences between conditions or groups. For the AMMT Oddball, a two‐tailed paired‐samples permutation *t*‐test between standard and deviant waveforms was conducted within each group to confirm the presence of the MMN. An independent samples permutation *t*‐test was conducted to examine group effects on the difference waveforms. ERP analyses were performed using BESA Statistics (version 2.1; MEGIS GmbH, Gräfelfing, Germany), which performs cluster‐based permutation testing and topographic ANOVAs that account for all electrodes and time points within an epoch, with robust correction for multiple comparisons.

Each permutation test proceeded in two stages. In the first stage, parametric *t*‐tests were computed at every electrode and time point: paired‐samples *t*‐tests for amplitude waveforms (e.g., AMMT Oddball standard vs. deviant trials; for the MST test phase, Target vs. Foil and FA Lure vs. Foil contrasts) and independent‐samples *t*‐tests for group comparisons of difference waveforms and CLARA source images (for the AMMT, the standard‐deviant difference; for the MST, the difference waveform between Target and Foil conditions, and between FA Lure and Foil conditions). Data points with *p*‐values below an initial cluster‐forming alpha threshold were grouped into spatiotemporal clusters. For the AMMT Oddball, the cluster alpha was set at 0.01, given the focal nature of the effects; for the MST Test phase, this was set at 0.05, given more distributed old–new effects. The *t*‐values within each cluster were then summed to generate a cluster‐level test statistic. In the second stage, Monte Carlo permutation testing was performed by randomly shuffling condition or group labels across 5000 iterations to generate a null distribution of maximum cluster‐level statistics under the assumption of exchangeability (i.e., no difference between conditions or groups). The observed cluster‐level statistics were then compared with this distribution to compute Monte‐Carlo *p*‐values, defined as the proportion of permutations yielding cluster values greater than or equal to the observed cluster value; for further details on cluster‐based permutation testing, see Maris and Oostenveld (2007). For the AMMT Oddball, permutation testing of difference waveforms was followed by additional within‐group permutation tests (i.e., standard vs. deviant waveforms within each group) to confirm the presence of the MMN and LDN.

Permutation testing was followed up by conventional ROI‐based approaches using a specified cluster of electrodes. For the AMMT Oddball phase, MMN amplitudes were extracted from seven frontocentral electrodes (i.e., AFz, F1, Fz, F2, FC1, FCz, and FC2) between 200 and 400 ms, and LDN amplitudes were extracted from 550 to 700 ms following Chow et al. (2025). To explore whether the sensory components of ERPs differ between groups, latencies and amplitudes of early auditory‐evoked responses (i.e., N1 and P1 components) from the AMMT Oddball were also examined only from standard trials. Peak latencies were measured as the maximum positivity or negativity within a specific time window. Upon visual inspection of grand mean waveforms, both P1 and N1 components appeared maximal at frontal and frontocentral sites and were thus extracted from the same cluster of seven frontocentral electrodes. Time windows for peak latencies were liberal (i.e., wider) to account for individual variability in latency and waveform skew, whereas time windows for mean amplitudes were more conservative (i.e., narrower) to reduce contamination from adjacent components, given individual variability in morphology and width of P1 and N1 components. P1 peak latencies were exported from each participant at the latency of the maximal positive‐going peak between 20 and 80 ms. N1 peak latencies were measured for each participant between 60 and 140 ms. As for amplitudes, P1 mean amplitudes were derived from the same electrode cluster between 30 and 70 ms, and N1 mean amplitudes between 80 and 120 ms.

For the MST Test phase, permutation testing was conducted within each group between Target and Foil conditions (i.e., Target–Foil contrast) to verify the presence of the FN400 and between groups to examine whether this component was modulated by musicianship. Permutation testing was also conducted on the difference between the FA Lure condition and the Foil condition (i.e., Lure–Foil contrast) to examine the group difference in the FN400 for false recognition of lures.

#### 2.8.2 Behavioral Measures

##### 2.8.2.1 AMMT Test Phase Accuracy

A generalized linear mixed‐effects model (GLMM) with a binomial logistic link was used to examine whether musicians showed enhanced performance on the test phase of the AMMT compared with nonmusicians. This approach was chosen to account for trial‐by‐trial variability between participants in addition to the primary effects of interest (i.e., Group and Condition). Fixed effects included Group (Musician, Nonmusician), Condition (Target, Lure, Foil), Trial (i.e., Trials 1 through 60), Age, and Group by Condition and Group by Trial interaction terms. For the Group factor, the nonmusician group was set as the reference level; for Condition, Target was used as the reference level, such that all Condition effects compared the accuracy of foils or lures relative to target trials. This structure allowed for examining group differences for the target–lure contrast (as an indication of mnemonic discrimination) and target–foil contrast (as an indication of the old/new effect in episodic memory). The fixed effects of Trial and Age were each standardized to facilitate convergence. Adjusted generalized variance inflation factors (GVIF^1/(2 × Df)^) were inspected to assess multicollinearity, and all adjusted GVIF values for fixed effects were below 2.5, indicating no serious multicollinearity among predictors. The initial model included random intercepts by participant to capture individual differences in overall baseline performance; a second model included random slopes for Condition to account for individual differences in response accuracy between stimulus types (e.g., some participants showing greater difficulty with lures than others). The *bobyqa* optimizer further facilitated convergence with an increased iteration limit of 200,000. Given that an exploratory model that included correlated random slopes for Condition failed to converge, uncorrelated slopes were specified to allow participant‐level variability in condition effects without estimating their correlation with the intercept. The model with uncorrelated random slopes for Condition provided a significantly better fit to the data than the intercept‐only model, *χ* ^2^(6) = 242.84, *p* < 0.001. Model fit indices also supported the second, more complex model (AIC = 3206.0, BIC = 3302.1) over the simpler intercept‐only model (AIC = 3436.9, BIC = 3496.9), and the log‐likelihood improved from −1708.4 (intercept‐only model) to −1587.0 (uncorrelated random slopes model); therefore, the final model included uncorrelated random slopes for Condition. Post hoc comparisons were conducted using estimated marginal means with Tukey adjustment for multiple comparisons. Simple effects analyses were performed to probe significant Group by Condition interactions.

##### 2.8.2.2 AMMT Interference Effects Over Time

To further examine the extent to which musicians show greater resistance to memory interference than nonmusicians, changes in accuracy over time on task (i.e., cumulative accuracy) were examined for each stimulus type using linear mixed‐effects modeling. This model included fixed effects of Group, stimulus type (i.e., Standard, Deviant, Lure1, Lure2, Lure3, and Foil), and Probe Position (i.e., from first to tenth position of each stimulus type across time on task). To account for baseline individual differences in trial‐wise performance across participants, model comparison was conducted to determine whether including random slopes for Probe Position significantly improved model fit. The initial model included random intercepts for each participant, whereas a more complex model included both random intercepts and random slopes for Probe Position within participants, specified as uncorrelated random effects. The more complex model with random slopes showed better fit than the intercept‐only model, *χ* ^2^(1) = 106.69, *p* < 0.001; in other words, accounting for individual differences in learning trajectories significantly improved the model's explanatory power. Simple slopes were used to examine significant three‐way interactions. In this context, steeper slopes denote a greater rate of cumulative accuracy across the 10 probe repetitions.

##### 2.8.2.3 Sensitivity Metrics

For the AMMT Test phase, *d*‐primes and criterion *c* values for each target–lure and target–foil contrast were subjected to one‐way ANCOVAs, with age and sex as covariates. Partial generalized eta‐squared was reported as a measure of effect size. Hit rates from all three conditions of the AMMT Perceptual Discrimination Task violated normality due to ceiling effects (identical pairs: *W* = 0.69, *p <* 0.001; for similar pairs: *W* = 0.87, *p <* 0.001; for dissimilar pairs: *W* = 0.51, *p <* 0.001). Therefore, nonparametric Mann–Whitney *U*‐tests were used to analyze auditory discrimination abilities between groups, with rank‐biserial correlations reported as effect size measures. As for the MST Test phase, LDI and old–new recognition performance were each subjected to the same aforementioned ANCOVA structures with age and sex as covariates.

### 2.9 Correlation and Mediation Analyses

Bivariate two‐tailed Pearson correlations with age as covariates were conducted to examine the relationship between ERPs and behavioral performance (i.e., MMN with AMMT *d*‐primes). Analyses were run with 1000 bootstrap samples, and bias‐corrected and accelerated (BCa) confidence intervals are reported. For variables that violated normality, Spearman rho correlations were also run while controlling for age, with 95% confidence intervals reported.

Mediation models using the R *lavaan* package were tested to examine whether MMN amplitude or perceptual discrimination ability mediated the effect of musicianship on AMMT target–lure and target–foil *d‐*primes. A series of parallel mediation models was specified, in which Group (Musician, Nonmusician) was entered as the exogenous predictor, *d*‐prime as the outcome variable, and MMN amplitude and perceptual discrimination hit rates were independent mediators. Specifically, for the target–lure discrimination model, the mediator was the hit rate for correctly identifying similar pairs; for the target–foil discrimination model, the mediator was the hit rate for correctly identifying dissimilar pairs. Models were estimated using full‐information maximum likelihood, and standard errors for indirect effects were estimated using 5000 bootstrap samples. All preprocessing and statistical analyses for behavioral measures were conducted using JASP (version 0.19.3) and R software (version 4.2.3).

## 3 Results

### 3.1 Neuropsychological Measures

Demographic and neuropsychological data for each group are presented in Table 1. Both groups were, on average, highly educated and showed average to high scores on general intellectual functioning, episodic memory, language, and executive functioning. For neuropsychological tests with scaled scores, nonparametric tests of significance (i.e., Bayesian Mann–Whitney *U*‐tests) were conducted. Analyses showed weak evidence of higher scaled scores on a test of processing speed (i.e., WAIS‐III Digit Symbol) for musicians than nonmusicians (*U* = 440.0, *p* = 0.029, *r* = 0.354, *B*_10_ = 1.37). Analyses on all other neuropsychological measures showed weak or null evidence of group differences. Bayesian independent‐samples *t*‐tests showed strong evidence for a group difference on the MET. As expected, musicians showed higher scores for the MET total score than nonmusicians, indicative of higher musical aptitude (*t*(49) = 3.20, *p* = 0.002, *d* = 0.90, *B*_10_ = 15.11). Musicians also scored higher for the melodic subdomain (*t*(49) = 3.29, *p* = 0.002, *d* = 0.92, *B*_10_ = 18.41). As for the rhythmic subdomain, analyses showed weak evidence for a group difference (*t*(49) = 2.28, *p* = 0.027, *d* = 0.67, *B*_10_ = 2.24).

**TABLE 1:** Participant demographic and neuropsychological data.

Variable	Older musician means (SD) *n* = 26		Older nonmusician means (SD) *n* = 25		*B*_10_
	Raw	Scaled	Raw	Scaled	
Demographics					
Age (years)	72.08 (6.58)	—	71.16 (6.08)	—	0.313
Education (years)	17.73 (2.51)	—	16.60 (2.47)	—	0.819
Sex (F:M)	16:10	—	13:12	—	0.426
TICS‐m	37.19 (2.65)	—	36.56 (3.70)	—	0.344
MoCA	27.46 (1.86)	—	27.20 (1.53)	—	0.360
biPTA threshold (dB HL)	18.82 (8.08)	—	19.61 (7.28)	—	
Musical abilities					
MET Melody	39.54 (5.38)	—	34.92 (4.62)	—	18.41^a^
MET Rhythm	39.88 (4.50)	—	36.80 (5.16)	—	2.43
MET Total	79.42 (8.40)	—	71.72 (8.78)	—	15.11^a^
Estimates of IQ					
WASI Vocabulary	67.08 (5.62)	13.19 (1.83)	66.12 (8.59)	12.60 (2.36)	0.424
WASI matrix reasoning	27.15 (2.87)	15.12 (1.63)	26.60 (2.42)	14.72 (1.46)	0.394
Memory					
CVLT‐3 Learning	44.19 (10.15)	10.69 (2.65)	46.80 (11.63)	11.36 (3.15)	0.353
CVLT‐3 Short‐Delay FR	9.81 (3.49)	11.38 (3.36)	9.60 (3.67)	11.04 (2.26)	0.296
CVLT‐3 Long‐Delay FR	10.42 (3.40)	11.12 (3.05)	10.16 (3.26)	10.80 (2.96)	0.297
CVLT‐3 recognition discriminability	2.94 (0.81)	11.27 (3.12)	2.76 (0.67)	10.56 (2.42)	0.393
WAIS‐III Digit Symbol IL FR	7.38 (1.17)	9.96 (1.28)	7.48 (1.08)	10.20 (1.08)	0.295
WAIS‐III Digit Symbol IL PR	11.85 (4.07)	10.27 (1.51)	13.20 (4.36)	10.60 (0.87)	0.355
Language					
D‐KEFS VF Letter Fluency	47.58 (11.42)	13.96 (2.88)	51.08 (13.32)	14.60 (3.59)	0.344
D‐KEFS VF Category Fluency	44.96 (8.58)	14.42 (3.06)	43.48 (6.47)	13.92 (2.48)	0.288
D‐KEFS VF Category Switching	14.12 (3.54)	12.62 (4.09)	14.52 (3.11)	12.88 (3.67)	0.286
Executive functioning, attention, processing speed					
WAIS‐III Digit Symbol‐Coding	73.23 (13.17)	14.46 (2.16)	66.68 (12.42)	13.16 (2.25)	1.68
WAIS‐IV Digit Span Forward	10.73 (2.20)	11.38 (2.97)	10.56 (2.33)	11.16 (2.95)	0.289
WAIS‐IV Digit Span Backward	9.15 (2.07)	11.62 (2.53)	9.64 (1.93)	12.20 (2.38)	0.377
WAIS‐IV Digit Span Sequencing	9.12 (1.68)	12.35 (2.38)	9.48 (2.26)	12.68 (2.82)	0.306
Trail Making Test A	23.42 (7.90)	14.73 (2.63)	25.36 (9.38)	14.08 (3.17)	0.347
Trail Making Test B	55.08 (22.61)	14.23 (3.02)	63.40 (25.49)	13.48 (2.80)	0.397
Mood					
GAD‐7	1.23 (1.21)	—	1.08 (1.73)	—	0.385
PHQ‐8	1.77 (2.12)	—	1.44 (1.61)	—	0.324

Abbreviations: *B*_10_ = Bayes factor for independent‐samples *t‐*tests or Mann‐Whitney *U* tests performed on scaled scores when available; biPTA = averaged bilateral pure‐tone audiometric threshold from 250 to 2000 Hz; CVLT‐3 = California Verbal Learning Test, 3rd edition; D‐KEFS = Delis–Kaplan Executive Functioning System; dB HL = decibels Hearing Level; FR = Free Recall; GAD‐7 = Generalized Anxiety Disorder 7‐item scale; IL = Incidental Learning; MET = Musical Ear Test; MoCA = Montreal Cognitive Assessment; PHQ‐8 = Patient Health Questionnaire 8‐item scale; PR = Paired Recall; TICS‐m = modified Telephone Interview of Cognitive Status; VF = Verbal Fluency; WAIS‐III, WAIS‐IV = Wechsler Adult Intelligence Scale, 3rd edition, 4th edition; WASI = Wechsler Abbreviated Scale of Intelligence.

^a^ Indicates strong evidence in favor of the alternative hypothesis.

### 3.2 Auditory MMN Memory Paradigm

#### 3.2.1 ERPs

##### 3.2.1.1 P1 and N1 Components

**FIGURE 1:**
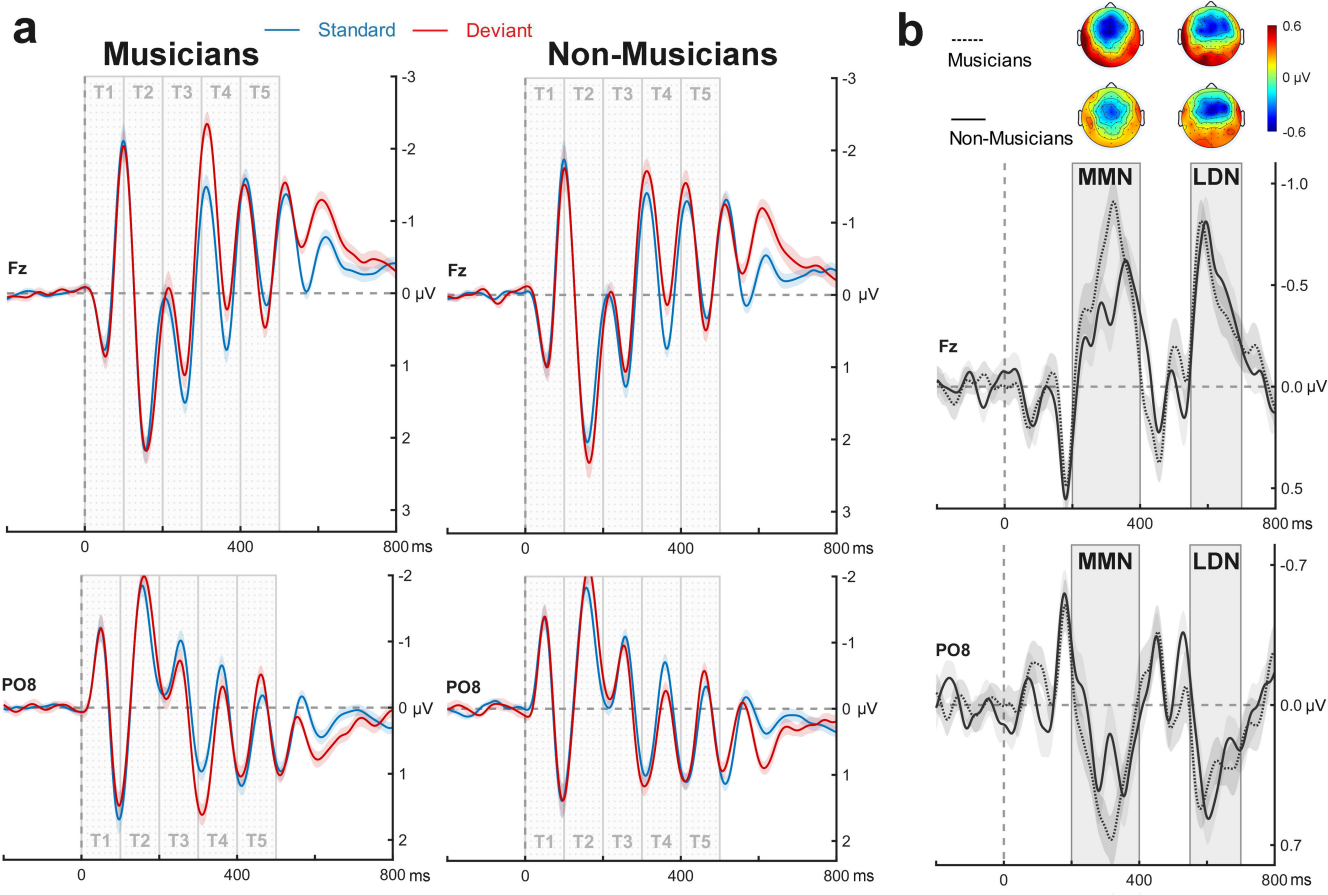
(a) Grand‐averaged standard and deviant waveforms of the oddball phase of the AMMT for musicians (*n* = 26) and nonmusicians (*n* = 25). P1 and N1 components are identified. Waveforms at the frontocentral midline (Fz) and right lateral parietal‐occipital electrode sites (PO8) show deviance‐related difference. (b) Difference waveforms and scalp topographies for the MMN (averaged between 200 and 400 ms) and LDN (averaged between 550 and 700 ms) for each group. Shaded regions represent time windows for the MMN and LDN shown over the Fz site; waveforms at the PO8 site show the polarity reversal of both components.

Standard and deviant grand‐averaged waveforms for both groups and the resulting difference waveforms and scalp topographies are displayed in Figure 1. As expected, the P1‐N1‐P2 complex generated by the first tone of the five‐tone sequence was visible and is a hallmark of the frontocentral auditory‐evoked potential (Picton 2010). The N1 corresponding to each of the four subsequent tones was also visible in both standard and deviant waveforms across musician and nonmusician groups. The ANCOVAs on early sensory components of the AEP (P1 and N1 of the P1‐N1‐P2 complex) with age and sex as covariates revealed no group difference for P1 latencies, *F*(1, 47) = 2.26, *p* = 0.139, *η* ^2^ *G* = 0.056, or for N1 latencies, *F*(1, 47) = 0.20, *p* = 0.659, *η* ^2^ *G* = 0.011. Similarly, analyses for mean amplitudes showed no group difference for either the P1 (*F*(1, 47) = 1.08, *p* = 0.305, *η* ^2^ *G* = 0.021) or N1 deflections (*F*(1, 47) = 0.51, *p* = 0.477, *η* ^2^ *G* = 0.013).

##### 3.2.1.2 MMN and LDN Components

The presence of MMN and LDN in each group was statistically verified using a data‐driven approach, followed by traditional analyses on mean amplitudes over a specified cluster of seven electrodes. The MMN was identified in both musician and nonmusician groups as an early frontal‐frontocentral negativity (approximately 200–400 ms) in deviant trials compared with standard trials. Permutation testing verified the presence of the MMN at frontal and frontocentral electrodes, as well as its polarity reversal that spanned lateral and temporal electrodes. The LDN was also visualized via a later frontal negativity (~500–750 ms) in deviant trials compared with standard trials. Similarly, permutation testing statistically quantified the presence of the LDN at frontal and frontocentral electrodes and its polarity reversal that spanned lateral and temporal electrodes. To statistically quantify the group difference in MMN amplitude, permutation testing was also conducted on the difference waveforms between musician and nonmusician groups. Analyses showed larger MMN amplitudes in musicians than nonmusicians (*p* = 0.044) at frontal and frontocentral electrodes. No clusters were identified as statistically significant between groups for the LDN. Cluster‐based statistics for the within‐group and between‐group comparisons are shown in Table 2.

**TABLE 2:** Summary of cluster‐based statistics: Standard vs. deviant waveforms of the AMMT oddball phase.

Electrode cluster		Channel at max	Max *t*‐value	Time (ms)	*p*
Older musicians					
1	FP1, AF7, AF3, F1, F3, F5, F7, FC5, FC3, FC1, C1, C3, CP1, CPz, FPz, FP2, AF8, AF4, AFz, Fz, F2, F4, F6, FC6, FC4, FC2, FCz, Cz, C2, C4, CP2	FC1	8.92	213–393	< 0.001
2	T7, TP7, P3, P5, P7, P9, PO7, PO3, O1, Iz, Oz, POz, FT8, C6, T8, TP8, CP6, P4, P6, P8, P10, PO8, PO4, O2, PO9, PO10, TP9, TP10, FT10	PO7	−7.39	225–424	< 0.001
3	AF7, AF3, F1, F3, F5, FC5, FC3, FC1, C1, C5, FPz, FP2, AF8, AF4, AFz, Fz, F2, F4, F6, F8, FT8, FC6, FC4, FC2, FCz, Cz, C2, C4	F2	8.52	551–713	< 0.001
4	T7, TP7, CP5, P3, P5, P7, P9, PO7, PO3, O1, Iz, Oz, POz, TP8, P6, P8, P10, PO8, O2, PO9, PO10, TP9, TP10	P5	−6.01	549–697	< 0.001
5	F1, F3, FC3, FC1, C1, C3, AF8, AF4, AFz, Fz, F2, F4, F6, F8, FC6, FC4, FC2, FCz, Cz, C2, C4, C6	FC4	−6.14	162–193	< 0.001
6	T7, TP7, P7, P9, PO7, O1, Iz, Oz, POz, P8, P10, PO8, PO4, O2, PO9, PO10, TP9, TP10	P10	5.90	166–193	< 0.001
7	F1, F3, FC3, FC1, C1, C3, AF8, AF4, AFz, Fz, F2, F4, F6, FC4, FC2, FCz	F1	−6.24	430–469	0.003
Older nonmusicians					
1	FP1, AF7, AF3, F1, F3, F5, FC5, FC3, FC1, C1, C3, FPz, FP2, AF8, AF4, AFz, Fz, F2, F4, F6, F8, FC6, FC4, FC2, FCz, Cz, C2, C4	F2	8.33	543–689	< 0.001
2	C5, T7, TP7, CP5, CP3, P1, P3, P5, P7, P9, PO7, PO3, O1, Iz, Oz, POz, Pz, C4, C6, T8, TP8, CP6, P6, P8, P10, PO8, PO4, O2, PO9, PO10, TP10	P8	−6.66	516–648	< 0.001
3	FP1, T7, TP7, P5, P7, P9, PO7, PO3, O1, Iz, Oz, FPz, FP2, AF8, AF4, AFz, F2, F4, F6, F8, FT8, FC4, FC2, C2, C4, C6, T8, TP8, P6, P8, P10, PO8, O2, PO9, PO10, TP10, FT10	T7	−5.15	324–469	< 0.001
4	C1, TP7, CP1, P1, P3, P5, P7, P9, PO7, PO3, O1, Iz, Oz, POz, Pz, CPz, FCz, Cz, TP8, P2, P4, P8, P10, PO8, PO4, O2, PO9, PO10, TP9, TP10	PO10	7.30	160–244	< 0.001
5	FP1, AF7, AF3, F1, F3, F5, FC5, FC3, FC1, C1, C3, CP1, CPz, FPz, FP2, AF8, AF4, AFz, Fz, F2, F4, F6, F8, FT8, FC6, FC4, FC2, FCz, Cz, C2, C4, C6	F4	−11.16	160–209	< 0.001
6	FP1, AF3, F1, F3, F5, FC5, FC3, FC1, C1, CP1, CPz, FPz, FP2, AF8, AF4, AFz, Fz, F2, F4, F6, FC6, FC4, FC2, FCz, Cz, C2	F1	5.66	320–396	< 0.001
Difference waveforms					
1	FP1, AF3, F1, F3, FC3, FC1, FPz, FP2, AF4, AFz, Fz, F2, F4, FC2, FCz	AFz	−4.50	301–334	0.044

*Note:* Musicians: Clusters 1 and 2 identified the presence of the MMN and its polarity reversal; Clusters 3 and 4 identified the presence of the LDN and its polarity reversal, respectively; Clusters 5 and 6 identified pre‐MMN frequency‐specific amplitude differences between conditions in the latency of the Auditory P2 (see Figure 1); Cluster 7 identified a short frontocentral positivity that may represent the oddball P300. Nonmusicians: Clusters 1 and 2 identified the presence of the LDN and its polarity reversal, respectively; Clusters 3 and 4 identified the presence of the LDN and its polarity reversal, respectively; Clusters 4 and 5 identified pre‐MMN frequency‐specific amplitude differences between conditions in the latency of the Auditory P2 and its polarity reversal (see Figure 1); Clusters 3 and 6 identified the mid‐to‐late portion of the MMN. Difference waveforms: Cluster 1 identified the time window in which MMN amplitude was statistically significant between groups. Spurious spatiotemporal clusters are not reported.

Results of permutation testing were verified by an ANCOVA between groups for MMN amplitude values used in the correlation (i.e., values averaged over a 200‐ to 400‐ms time window, across seven frontal and frontocentral electrodes) with age and sex as covariates. As expected, musicians showed larger MMN amplitudes than nonmusicians, *F*(1, 47) = 5.57, *p* = 0.022, *η* ^2^ *G* = 0.098. An ANCOVA with the same covariates was performed for LDN amplitudes but showed no group difference, *F*(1, 47) = 0.25, *p* = 0.622, *η* ^2^ *G* = 0.003.

**FIGURE 2:**
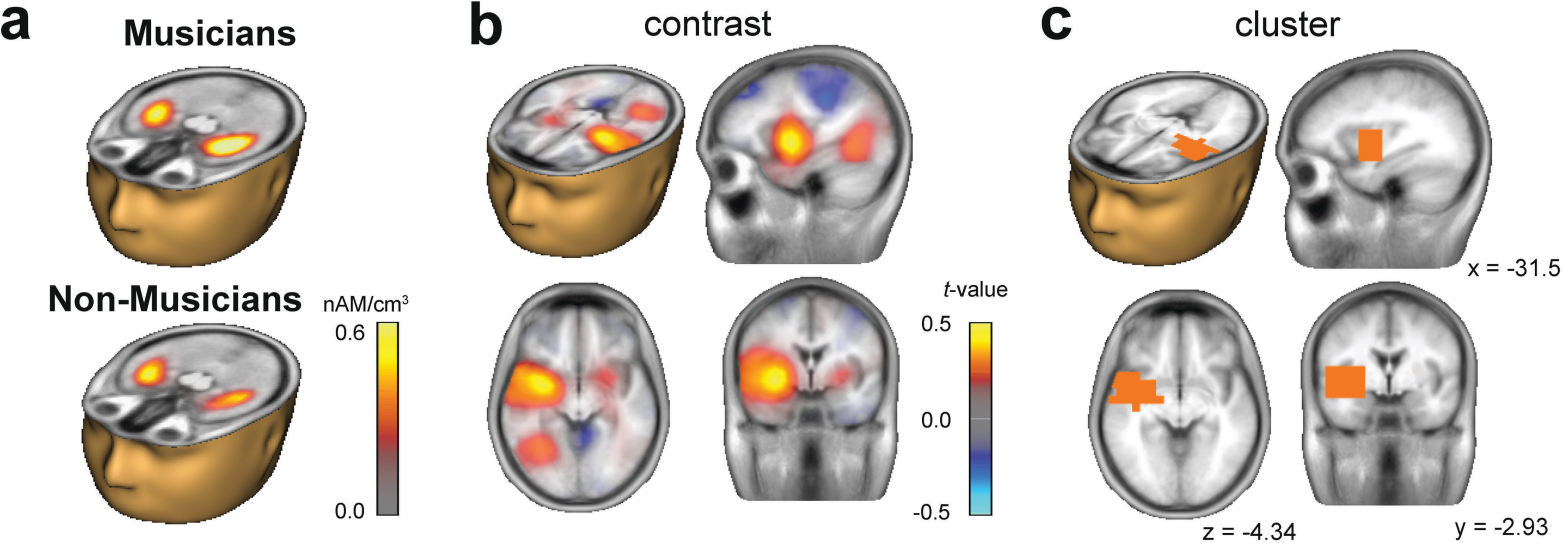
(a) Grand‐averaged source reconstructions for each group of the standard‐deviant difference waveform, averaged between a conservative 250‐ to 350‐ms time window. (b) *t*‐value contrast between groups showing left‐lateralized enhanced source activity in older musicians. (c) Spatial distribution of the cluster identified from permutation testing as statistically significantly between groups. Coordinates are reported in Talairach space.

CLARA MMN source reconstructions were averaged over a 250‐ to 350‐ms time window representing most of the deviant‐related response. Both groups showed focal bilateral source activation spanning primary auditory, superior temporal, and inferior frontal regions. Permutation testing revealed one cluster (maximum *t‐*value = 4.79, *p* = 0.006, statistical extrema at Talairach coordinates *x* = −31.5, *y* = −2.93, *z* = −4.34). This cluster is interpreted as greater source activity for older musicians compared with nonmusicians, likely encompassing the left A1, superior temporal gyrus, and inferior frontal gyrus (IFG) (Figure 2; though this localization is considered inferential given the distributed nature of CLARA images).

#### 3.2.2 Behavioral Measures: Test Phase

##### 3.2.2.1 Accuracy

**FIGURE 3:**
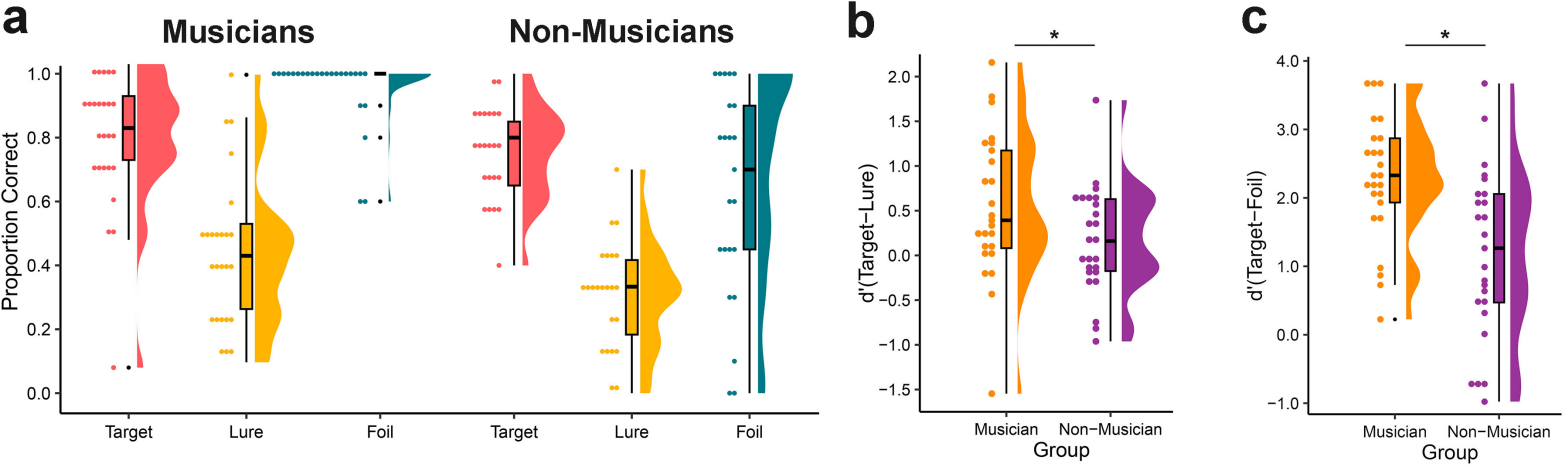
(a) Raincloud plots of accuracy on the Auditory MMN Memory Task by group and condition. (b) Raincloud plots of *d*‐primes on the Auditory MMN Memory Task by group for target–lure and (c) for target–foil discriminability.

Figure 3a shows accuracy per condition and group in the subsequent test phase. A generalized linear mixed model (GLMM) with a binomial distribution was used to examine trial‐level accuracy, with random intercepts and uncorrelated random slopes for Condition included for each participant; Table 3 shows a summary of model parameters. A significant Group by Condition interaction was found for foil accuracy compared with targets, *b* = 3.73, SE = 0.98, *z* = 3.79, *p* < 0.001. Post hoc comparisons indicated that musicians were more accurate than nonmusicians for foils, *b* = −3.91, SE = 0.98, *z* = −3.99, *p* < 0.001, whereas target accuracy did not differ between groups, *b* = −0.19, SE = 0.30, *z* = −0.61, *p* = 0.540. However, the Group by Condition interaction for the target–lure contrast was not significant, *b* = 0.23, SE = 0.51, *z* = 0.45, *p* = 0.653, indicating that the reduction in accuracy for lures (relative to targets) did not differ between groups.

**TABLE 3:** GLMM parameters of AMMT Test phase hit rates.

Predictor	Estimate (*b*)	SE	*z*	*p*
Intercept	1.21	0.21	5.68	< 0.001**
Group	0.19	0.30	0.61	0.540
Condition (foil vs. target)	0.02	0.56	0.04	0.966
Condition (lure vs. target)	−2.08	0.36	−5.82	< 0.001
Trial	−0.14	0.06	−2.32	0.021*
Age	0.07	0.09	0.76	0.447
Group × Condition (foil)	3.73	0.98	3.79	< 0.001***
Group × Condition (lure)	0.23	0.51	0.45	0.653
Group × Trial	−0.14	0.09	−1.48	0.138

*Note:* Nonmusician was set as the reference for Group, and Target was set as the reference for Condition. Trial (1–60) and age variables were *z*‐scored.

As for Condition effects (collapsing across groups), the analysis revealed worse accuracy for lures compared with targets as expected, *b* = −2.08, SE = 0.36, *z* = −5.82, *p* < 0.001, with no significant difference between foil and target accuracy, *b* = 0.02, SE = 0.56, *p* = 0.966. Within‐group comparisons indicated that, for nonmusicians, accuracy for lures was lower than that of foils (*b* = 2.10, SE = 0.61, *z* = 3.45, *p* = 0.002) and targets (*b* = 2.08, SE = 0.36, *z* = 5.82, *p* < 0.001), with no difference between foil and target accuracy (*b* = −0.02, SE = 0.56, *z* = −0.04, *p* = 0.999). Similarly, within musicians, accuracy for lures was lower than for foils (*b* = 5.60, SE = 0.92, *z* = 6.08, *p* < 0.001) and for targets (*b* = 1.85, SE = 0.36, *z* = 5.13, *p* < 0.001); however, accuracy was higher for foils than targets (*b* = −3.75, SE = 0.89, *z* = −4.21, *p* < 0.001).

The main effect of stimulus type reached significance, indicating a slight decline in accuracy over time on the task, *b* = −0.14, SE = 0.06, *p* = 0.021, though this did not vary by group (i.e., no Group by Trial interaction, *b* = −0.14, SE = 0.09, *z* = −1.48, *p* = 0.138). The effect of Group was nonsignificant, *b* = 0.19, SE = 0.30, *p* = 0.54, and the effect of Age was also not significant, *b* = 0.07, SE = 0.09, *p* = 0.447. In summary, accuracy for lures was significantly worse than for targets or foils within each group, as expected. Musicians demonstrated less interference from foil probes than nonmusicians; no group difference was observed for target or lure accuracy.

##### 3.2.2.2 Interference Effects Over Time

Table 4 shows a summary of model parameters from the linear mixed model (LMM) that examined cumulative accuracy for each of the six memory probes (i.e., Stimulus Type) and its relative position in the task (i.e., Probe Position from 1 to 10) as a measure of resistance to interference over time. As expected, the main effect of Probe Position was significant, *b* = 0.78, SE = 0.04, *t* = 19.86, *p* < 0.001, indicating that cumulative accuracy improved overall across trials. The model also showed a significant two‐way interaction between Probe Position and Stimulus Type; compared with the Standard probe, the rate of cumulative hits over time on task was slower for all three lure types, as expected (Lure 1, *b* = −0.50, *p* < 0.001; Lure 2, *b* = −0.44, *p* < 0.001; and Lure 3, *b* = −0.49, *p* < 0.001). Notably, the model revealed a significant three‐way interaction between Group, Stimulus Type, and Probe Position for cumulative accuracy for the Foil probe (relative to Standard), *b* = 0.24, SE = 0.066, *t* = 3.59, *p* < 0.001. Simple slopes analyses revealed, for the Foil probe, steeper cumulative accuracy rates for older musicians compared with nonmusicians, *b* = 0.30, SE = 0.06, *t*(397) = 5.45, *p* < 0.001. No other group differences in cumulative accuracy rates reached significance (relative to Standard, Deviant: *b* = −0.07, *p* = 0.230; Lure 1: *b* < 0.01, *p* = 0.971; Lure 2: *b* = −0.04, *p* = 0.595; Lure 3: *b* = 0.03, *p* = 0.608). In summary, the model demonstrated that overall cumulative accuracy increased with probe repetition. As expected, the model showed slower cumulative accuracy over time (i.e., greater vulnerability to interference) for each lure probe relative to the Standard probe. Nonmusicians showed greater vulnerability to interference over time for foils than musicians.

**TABLE 4:** LMM results of AMMT Test phase cumulative hit rates per stimulus type.

Fixed effect	Estimate (*b*)	SE	*df*	*t*	*p*
Intercept (standard, nonmusician)	−0.229	0.204	2928	−1.13	0.260
Group (musician vs. nonmusician)	−0.077	0.288	2928	−0.27	0.788
Probe position	0.780	0.039	2928	19.86	< 0.001
Stimulus: Foil vs. standard	0.413	0.288	2928	1.44	0.151
Stimulus: Lure1 vs. standard	0.517	0.288	2928	1.80	0.073
Stimulus: Lure2 vs. standard	0.576	0.288	2928	2.00	0.046
Stimulus: Lure3 vs. standard	0.347	0.288	2928	1.20	0.229
Stimulus: Deviant vs. standard	0.141	0.288	2928	0.49	0.624
Group × Probe Position	0.067	0.056	420.5	1.21	0.227
Group × Stimulus: Foil	−0.051	0.407	2928	−0.12	0.901
Group × Stimulus: Lure1	0.315	0.407	2928	0.77	0.440
Group × Stimulus: Lure2	0.248	0.407	2928	0.61	0.543
Group × Stimulus: Lure3	0.443	0.407	2928	1.09	0.277
Group × Stimulus: Deviant	−0.075	0.407	2928	−0.18	0.855
Probe Position × Stimulus: Foil	−0.130	0.046	2928	−2.81	0.005
Probe Position × Stimulus: Lure1	−0.505	0.046	2928	−10.88	< 0.001
Probe Position × Stimulus: Lure2	−0.435	0.046	2928	−9.37	< 0.001
Probe Position × Stimulus: Lure3	−0.489	0.046	2928	−10.54	< 0.001
Probe Position × Stimulus: Deviant	−0.023	0.046	2928	−0.49	0.624
Group × Probe Position × Stimulus: Foil	0.235	0.066	2928	3.59	< 0.001
Group × Probe Position × Stimulus: Lure1	0.002	0.066	2928	0.04	0.971
Group × Probe Position × Stimulus: Lure2	−0.035	0.066	2928	−0.53	0.595
Group × Probe Position × Stimulus: Lure3	0.034	0.066	2928	0.51	0.608
Group × Probe Position × Stimulus: Deviant	−0.079	0.066	2928	−1.20	0.230

*Note:* Nonmusician was set as the reference for Group, and Standard was set as the reference for Stimulus.

##### 3.2.2.3 Sensitivity Metrics

Results from the ANCOVA on *d*‐primes showed that musicians outperformed nonmusicians on target–lure discriminability, *F*(1, 46) = 4.27, *p* = 0.045, *η* ^2^ *G* = 0.076 (see Figure 3b,c). Furthermore, musicians also outperformed nonmusicians on target–foil discriminability, *F*(1, 46) = 13.87, *p <* 0.001, *η* ^2^ *G* = 0.219. As for response bias (*c*), older musicians adopted a more conservative decision criterion (greater values for *c*) for target–foil discriminability compared with nonmusicians, *F*(1, 46) = 12.40, *p* < 0.001, *η* ^2^ *G* = 0.007. In other words, nonmusicians were more likely to incorrectly endorse the foil probe as “old,” consistent with greater false alarm rates in this group than musicians. No group difference was found for response bias for target–lure discriminability, *F*(1, 46) = 0.37, *p* = 0.546, *η* ^2^ *G* = 0.200.

#### 3.2.3 Behavioral Measures: Perceptual Discrimination Task

**FIGURE 4:**
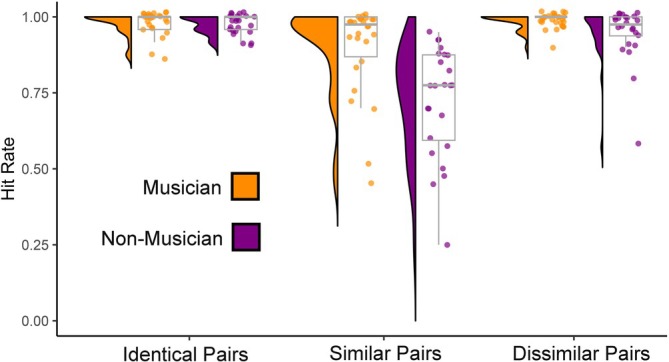
Hit rates of the AMMT Perceptual Discrimination Task by group, showing greater hit rates for discriminating between similar and dissimilar tone sequences in older musicians than nonmusicians.

Hit rates for the Perceptual Discrimination Task by group are shown in Figure 4. Mann–Whitney *U*‐tests revealed no group difference on hit rates for identifying identical pairs (*U* = 272.5, *z* = −0.89, *p* = 0.374, *r* = 0.127). In contrast, musicians showed on average greater accuracy for correctly discriminating similar pairs (*U* = 108.5, *z* = −3.97, *p* < 0.001, *r* = 0.652) and for discriminating between dissimilar pairs (*U* = 215.0, *z* = −2.21, *p* = 0.027, *r* = 0.312).

### 3.3 Correlation Analyses

Partial correlations controlling for the effects of age and sex showed that the relationship between MMN amplitude and target–lure *d*‐primes did not reach significance within musicians (*r*(22) = −0.003, *p* = 0.988, BCa CI [−0.262, 0.298]), within nonmusicians (*r*(22) = −0.260, *p* = 0.209, BCa CI [−0.553, 0.115]), or for the two groups combined (*r*(47) = −0.089, *p* = 0.539, BCa CI [−0.278, 0.127]). The relationship between MMN amplitude and target–foil *d*‐primes was also nonsignificant for within musicians (*r*(22) = −0.088, *p* = 0.674, BCa CI [−0.444, 0.264]), within nonmusicians (*r*(22) = −0.319, *p* = 0.120, BCa CI [−0.579, 0.023]), or for the two groups combined (*r*(47) = −0.104, *p* = 0.472, BCa CI [−0.373, 0.173]). In other words, individual differences in MMN amplitude in older musicians and nonmusicians did not explain sensitivity metrics in memory performance on the AMMT.

Additional post hoc correlation analyses with measures on the Perceptual Discrimination Task showed that, across the sample, greater MMN amplitude was associated increased hit rates for discriminating similar pairs (*ρ*(47) = 0.29, *p* = 0.040, 95% CI [0.04, 0.532]) while controlling for age, whereas no significant association was found for hit rates for the other two conditions (dissimilar pairs: *ρ*(47) = −0.13, *p* = 0.384, 95% CI [−0.408, 0.175]; for identical pairs: *ρ*(47) = −0.13, *p* = 0.377, 95% CI [−0.416, 0.156]). However, this association between MMN amplitude and discriminating similar pairs was not significant when split by group (Musicians: *ρ*(23) = 0.09, *p* = 0.653, 95% CI [−0.300, 0.496]; Nonmusicians: *ρ*(21) = 0.21, *p* = 0.337, 95% CI [−0.219, 0.607]).

### 3.4 Mediation Analyses

**FIGURE 5:**
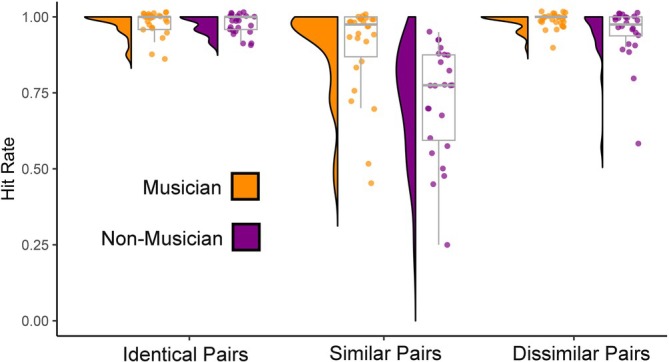
Schematic of parallel mediation models testing potential mediators of MMN amplitude and hit rates on the Perceptual Discrimination Task for (a) group differences on target–lure discriminability through discriminating similar pairs and (b) on target–foil discriminability through hit rates for dissimilar pairs. Standardized path coefficients are reported.

Mediation models were run to examine whether enhanced target–lure and target–foil discriminability in older musicians was explained by enhanced MMN amplitudes or perceptual discrimination abilities. A series of parallel mediation models were specified where Group (Musician, Nonmusician) was entered as the exogenous predictor, and *d*‐prime was entered as the outcome variable, and MMN amplitude and perceptual discrimination hit rates were independent mediators (see Figure 5 for a schematic of the model and results of model parameters). For the target–lure discrimination model, Group significantly predicted MMN amplitude, *B* = −0.20, SE = 0.09, *p* = 0.021, accounting for 10% of the variance (*R* ^2^ = 0.10). Group also significantly predicted discrimination of similar pairs, *B* = −0.17, SE = 0.05, *p* < 0.001, explaining 22% of the variance (*R* ^2^ = 0.22). The direct effect of Group on *d′*(T,L) was not significant, *B* = −0.30, SE = 0.21, *p* = 0.157, with MMN amplitude (*B* = −0.33, SE = 0.30, *p* = 0.276) and discrimination of similar items (*B* = 0.88, SE = 0.60, *p* = 0.142) also not significantly predictive of *d′*(T,L) values. Importantly, the Group effect on *d′*(T,L) was not significantly mediated by MMN amplitude, *B* = 0.065, SE = 0.07, *p* = 0.369, or through perceptual discrimination for similar pairs, *B* = −0.154, SE = 0.12, *p* = 0.192. The total effect of Group on *d′*(T,L) reached significance, *B* = −0.39, SE = 0.20, *p* = 0.047, *R* ^2^ = 0.13.

As for the target–foil discrimination model, Group significantly predicted MMN amplitude, *B* = −0.20, SE = 0.09, *p* = 0.022, accounting for 10% of the variance (*R* ^2^ = 0.10). Group also significantly predicted discrimination of dissimilar pairs, *B* = −0.04, SE = 0.02, *p* = 0.036, *R* ^2^ = 0.09. The direct effect of Group on *d′*(T,F) remained significant, *B* = −0.97, SE = 0.37, *p* = 0.010. However, MMN amplitude (*B* = 0.19, SE = 0.36, *p* = 0.590) and ability to discriminate dissimilar pairs (*B* = 2.42, SE = 2.55, *p* = 0.343) did not significantly predict *d′*(T,F). As for indirect effects, Group effects on *d′*(T,F) were not mediated by MMN amplitude, *B* = −0.04, SE = 0.08, *p* = 0.617, or through discrimination of different foils, *B* = −0.10, SE = 0.09, *p* = 0.236. The total effect of Group on *d′*(T,F) was significant, *B* = −1.11, SE = 0.31, *p* < 0.001, *R* ^2^ = 0.23.

### 3.5 MST

#### 3.5.1 ERPs

**FIGURE 6:**
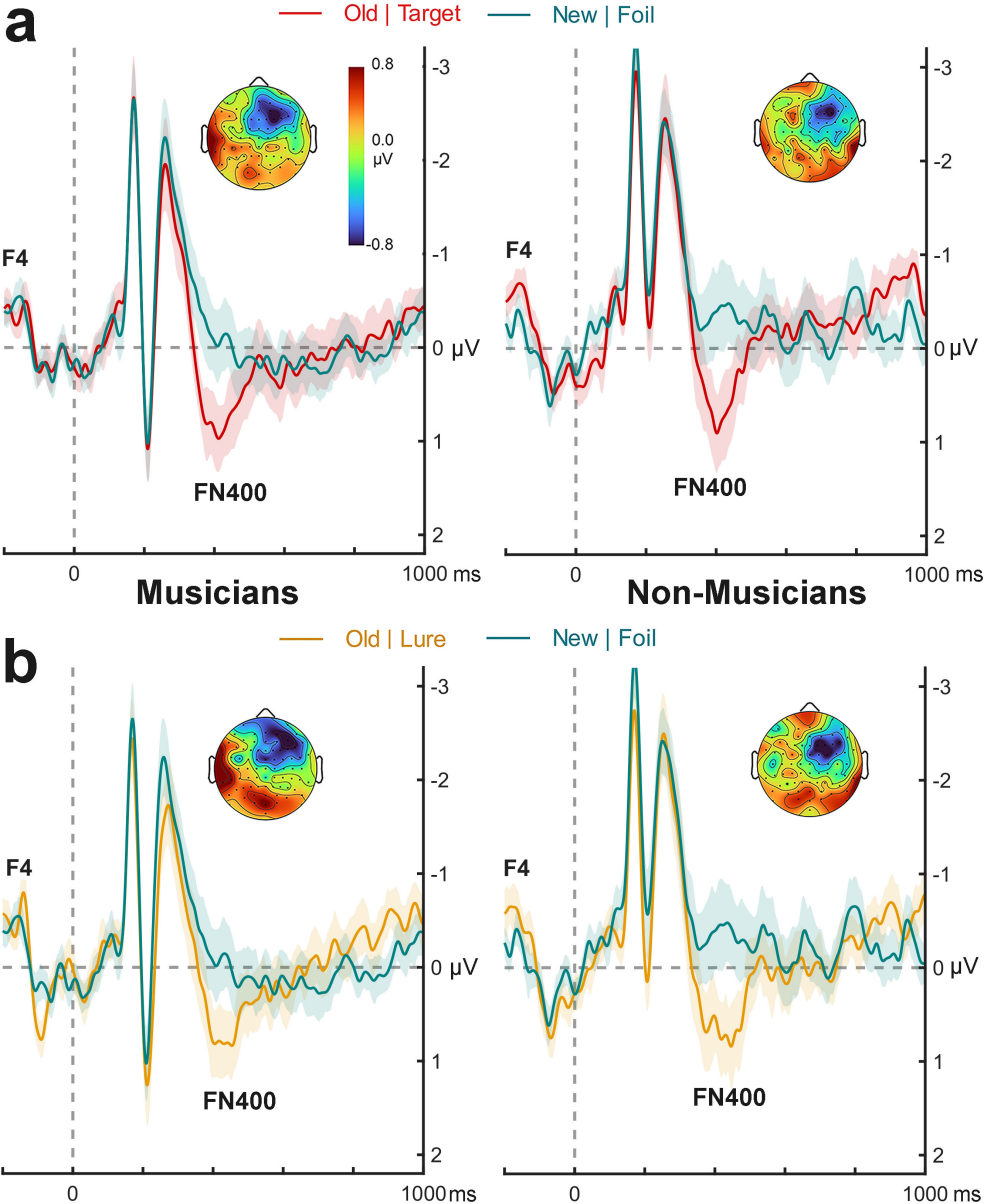
Grand‐averaged event‐related potentials of the MST Test phase by group; waveforms are shown from a representative right frontal electrode (F4). (a) Target–foil contrast with scalp topographies showcasing an FN400 modulation averaged over 400–600 ms; (b) false alarm lure–foil contrast with scalp topographies averaged over 450–650 ms.

ERPs of the test phase of the MST, including the resulting difference waveforms and scalp topographies, are displayed in Figure 6. Upon visual inspection, the P1‐N1 complex of the visual‐evoked potential was visible in both groups as expected. In the Target–Foil contrast, a subsequent old–new modulation was also visible in both groups over frontal and frontocentral scalp regions, with a greater negativity for foil than target trials, and was therefore identified as the FN400. Similarly, in the Lure–Foil contrast, the FN400 was also visible in both groups over frontal and frontocentral sites, with a greater negativity for foil than falsely recognized lures. Within each group, cluster‐based permutation testing confirmed the presence of the FN400 in both contrasts (see Supporting Information). However, no group difference was found in the FN400 modulation for either the Target–Foil or Lure–Foil contrast.

#### 3.5.2 Behavioral Performance

**FIGURE 7:**
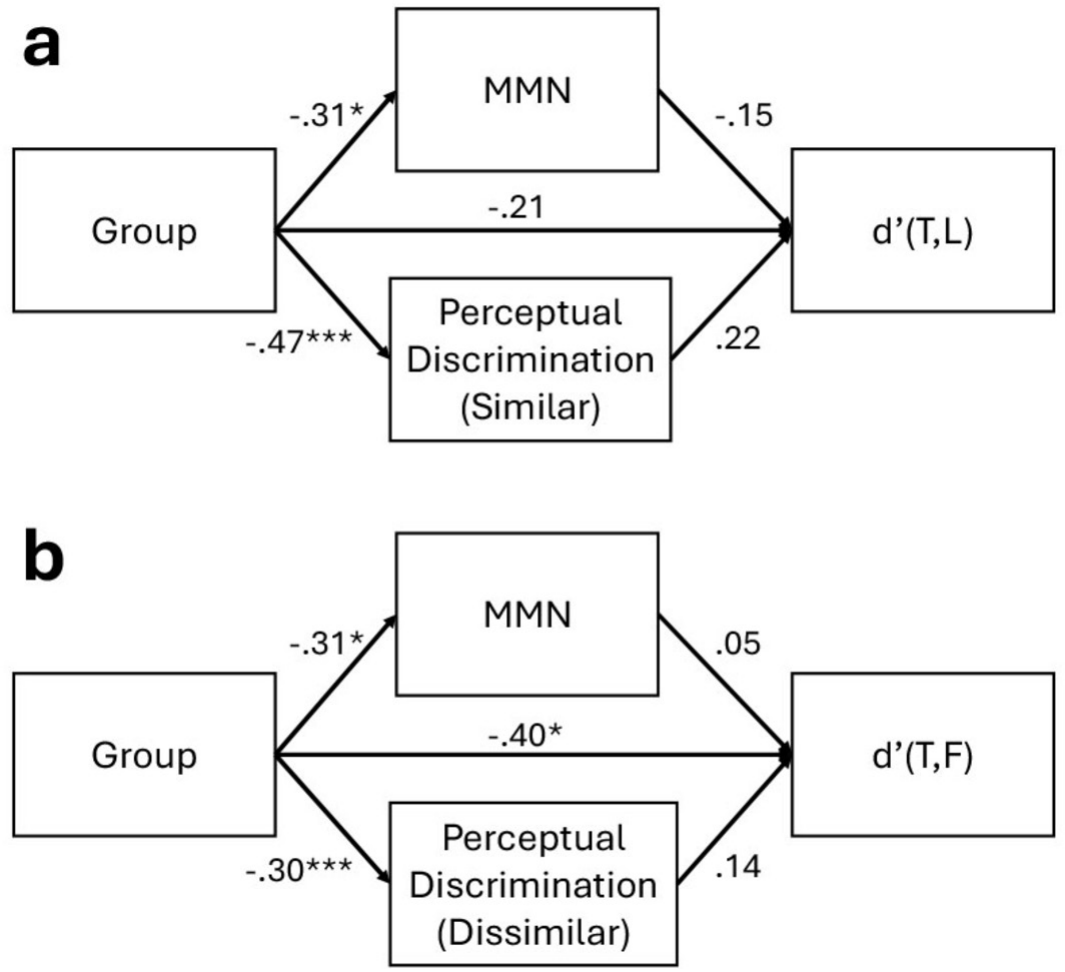
Raincloud plots of (a) lure discrimination index and (b) old–new recognition performance on the MST by group.

As shown in Figure 7, the group difference for LDI score did not reach significance, *F*(1, 45) = 0.09, *p* = 0.762, *η* ^2^ *G* = 0.003, BF_10_ = 0.302. Likewise, no group difference was shown for old–new recognition performance, *F*(1, 45) = 0.257, *p* = 0.615, *η* ^2^ *G* = 0.006, and moderate evidence for a null difference between groups (BF_10_ = 0.278).

### 3.6 Summary of Results

On the AMMT Oddball, older musicians showed enhanced MMN amplitudes and greater source activity spanning left IFG and auditory areas compared with nonmusicians. On the subsequent test phase, both groups were more likely to falsely endorse lures that were “old” compared with targets and foils in both groups, as expected. Sensitivity metrics revealed enhanced target–lure and target–foil *d*‐primes in musicians, indicative of enhanced memory precision for oddball stimuli and resistance to interference from similar lures and dissimilar foils. Nonmusicians showed greater false alarm rates for foils compared with targets (i.e., more likely to incorrectly endorse the foil probe as “old”) compared with musicians. When examining each probe type, results further showed (a) increased interference across repetitions for each lure type across groups and (b) greater interference from repetition of the foil probe over time in nonmusicians than musicians. No correlation was found between MMN and subsequent memory performance, though MMN amplitude across the older adult sample was correlated with the ability to discriminate similar tone sequences while accounting for age. Although musicians showed better perceptual discrimination between similar and dissimilar pairs of tone sequences, mediation analyses showed that this benefit did not explain the group differences on target–lure and target–foil *d‐*primes. No group differences were found for early auditory P1 or N1 components, or for the LDN. As for the MST, no group difference was observed for the FN400 component or for behavioral measures of lure discrimination or old/new recognition.

## 4 Discussion

Our results suggest that extensive musical engagement is associated with enhanced precision in auditory perception and memory in older adults. We observed enhanced MMN amplitudes in older musicians compared with nonmusicians, accompanied by increased source activity in left auditory and frontal regions for oddball detection. The MMN was selectively enhanced in older musicians, as no group differences were observed in earlier sensory components (i.e., P1 and N1) or in later deviance‐related potentials (i.e., the LDN). Notably, older musicians performed with higher accuracy on auditory lure and foil discrimination than their nonmusician counterparts. Furthermore, older musicians showed enhanced back‐to‐back perceptual discrimination abilities for similar and dissimilar tone sequences, which did not explain enhanced lure and foil discrimination. Although MMN in older adults was related to perceptual discrimination of similar tone sequences, this relationship did not differ across groups. Overall, these findings provide neural and behavioral evidence for enhanced, domain‐specific auditory processing in older musicians compared with nonmusicians, consistent with prior research in other auditory abilities such as speech‐in‐noise processing (Bidelman and Krishnan 2010; Parbery‐Clark, Skoe, and Kraus 2009; Parbery‐Clark, Skoe, Lam, and Kraus 2009; Zendel and Alain 2014). Additionally, findings indicate enhanced perceptual and mnemonic representations of auditory stimuli in older musicians that may contribute to cognitive reserve in aging.

### 4.1 Enhanced MMN Source Activity in Older Musicians

The source activity underlying enhanced amplitudes to the contour‐deviant MMN was greater in older musicians than in nonmusicians. These results suggest that long‐term engagement of musical activities is associated with enhanced auditory sensory memory, with enhancements observed even in older musicians using complex deviants (i.e., violations to higher‐order abstract regularities, or the relational structure across elements within auditory stimuli) (Avissar et al. 2018; Paavilainen et al. 2018; Paavilainen 2013; Salisbury 2012). Furthermore, enhanced source activity underlying the MMN was left lateralized to auditory and inferior frontal regions, consistent with prior studies positing a network of frontal and auditory sources for MMN generation (Opitz et al. 2002; Doeller et al. 2003). The conventional network implicated in MMN generation (e.g., to pitch deviants) has involved the right rather than the left IFG, in addition to the superior temporal gyri (Garrido et al. 2008; Garrido, Kilner, Kiebel, and Friston 2009; Opitz et al. 2002). However, more recent investigations have implicated the left IFG in the network underlying the generation of MMN to abstract, complex irregularities, such as omission deviants within expected tone sequences (Chennu et al. 2016; Phillips et al. 2015, 2016; see Heilbron and Chait 2018), which reflect higher‐order regularity violations, comparable with complex pitch contour violations used in the present study. One potential explanation that aligns with the predictive coding framework is that greater frontal source activity in musicians may reflect stronger violations from top‐down comparisons between incoming and predicted auditory inputs. In other words, musicians may indicate more robust internal predictive models of upcoming sensory events, due to stronger sensory memory traces of the standard stimulus (Garrido, Kilner, Kiebel, and Friston 2009; Garrido, Kilner, Stephan, and Friston 2009).

Regarding left‐lateralization of source activity, results also extend prior work demonstrating left‐lateralized MMN responses in young adult musicians, particularly with other similar complex deviants such as abstract violations of tone patterns (Herholz et al. 2009), timbre violations (Vuust et al. 2012), and meter and rhythmic deviants (i.e., relational hierarchy of strong and weak positions within a sequence; Vuust et al. 2005, 2009). It has been suggested that hemispheric lateralization reflects differences in processing specific auditory features (rather than music vs. speech per se), with the left hemisphere preferentially engaged by temporal information (such as change in pitch contour over time), and the right hemisphere more sensitive to spectral (pitch‐based) information (Tervaniemi 2026). Furthermore, the MMN to lexical tones was found to be right‐lateralized for pitch deviants and left‐lateralized for pitch‐contour deviants (Wang et al. 2013), consistent with oddball stimuli in the present study. Furthermore, our results are consistent with studies examining long‐term musical enculturation effects (Haumann et al. 2018), showing that long‐term listening to musical patterns may contribute to left lateralization akin to language‐based studies (Pulvermüller et al. 2006). In addition, multisensory studies show that musicians demonstrate higher MMN source activity in the left superior temporal gyrus during audiovisual nonsymbolic congruent trials and lower activation for incongruent trials compared with nonmusicians (Chalas et al. 2022). Furthermore, shifting of neural sources of the MMN has been highlighted as a hallmark of impaired sensory processing in many populations (e.g., in mild cognitive impairment; Papadaniil et al. 2016; Tsolaki et al. 2017). Collectively, these studies suggest that MMN source generation may reflect neuroplastic changes from active musical engagement.

Our study provides neural evidence for enhanced precision in auditory discrimination in older musicians. These findings are notable, particularly given robust age‐related declines in MMN amplitude between young and older adults (Cheng et al. 2013). Research further demonstrates that MMN amplitude is further attenuated for individuals with mild cognitive impairment (i.e., the prodromal stage of dementia) and early stages of Alzheimer's disease compared with healthy older adults (Lindín et al. 2013; Papadaniil et al. 2016; Tsolaki et al. 2017), with smaller MMN amplitudes related to worse neuropsychological performance for verbal learning (Mowszowski et al. 2012). Given that enhancements in MMN responses may be associated with preserved sensory processing in aging, it may be necessary to determine whether music training could insulate age‐related decreases in sensory processing through preserved encoding of auditory information. As prior work has suggested that instrumental music training may protect against dementia risk (e.g., Balbag et al. 2014; see Arafa et al. 2022 and Walsh et al. 2021 for review), future research might examine the role of music training in the maintenance of sensory health and whether preserved sensory function may mitigate downstream declines in specific cognitive domains with age. Results did not show a correlation between the MMN amplitude and subsequent memory performance. There are many potential explanations for these findings. First, although expertise in auditory tasks such as playing a musical instrument is associated with perceptual sensitivity (Candidi et al. 2014; Groussard et al. 2014; Pantev et al. 1998; Proverbio and Bellini 2018), one limitation of our research is the heterogeneity of the musician group, with musicians reporting a wide range of musical experiences. Each musical instrument requires different perceptual and motor abilities. Despite essential differences in sensorimotor performance, many studies, including the current study, place instrumentalists in one category, mixing professional string and wind players (Rodrigues et al. 2013); amateur instrumentalists (Bidelman and Alain 2015); or a mix of professional, amateur, and school‐recruited musicians (Yamashita et al. 2022). Although most studies report that their samples of musicians were actively playing an instrument at the time of testing, the extent to which these previous musical experiences dilute or confound perceptual processing is unknown. In addition, some musicians in the current study and other studies (Yamashita et al. 2022) reported practicing multiple instruments, which may increase variability in musical engagement activities that may underscore enhanced precision in perception. The age at which training commenced, type of training (method or approach) received (e.g., Suzuki Method and Orff Schulwerk approach), intensity of the training, and genre of specialized training (e.g., jazz, classical) could also affect perceptual processing (Bianco et al. 2018). Auditory cortical representations and responses have been associated with the years of music training and the age at which training commenced (Pantev et al. 1998). Additionally, the types of training employed and gene–environment interactions may account for auditory abilities (e.g., the degree to which home and academic/work environments reinforced the development of musical skills). Self‐report measures such as the Goldsmiths Musical Sophistication Index (Müllensiefen et al. 2014) only partially account for the role of home listening environments on perceptual processing; thus, there is a need for robust objective measures of musical experiences. Further studies examining perceptual processing and memory precision in musicians may consider including musical achievement measures to account for variability in musicianship (e.g., dabbler, amateur, and professional).

### 4.2 Mnemonic Representations in Older Musicians

Based on sensitivity metrics (i.e., *d*‐primes), the present study found evidence for enhanced mnemonic representations for auditory oddball stimuli in older musicians when presented with distractor lures and foils. These findings were notable, particularly given evidence for null differences between groups on baseline neuropsychological measures of episodic memory and auditory attention. Though these findings could be attributed to enhanced auditory discrimination abilities and the encoding of contour information in musicians (Zak et al. 2026), we view this possibility as unlikely, given that these effects in memory were not explained by perceptual discrimination ability as shown through mediation models. These enhancements to mnemonic representations were limited to the auditory domain, as no group differences on neural or behavioral measures on the visual MST were found in the present study. Notably, the visual MST differs substantially from the auditory paradigm in task stimuli (i.e., recognizable objects rather than abstract stimuli), structure (i.e., unique vs. repeated stimuli), and response format (i.e., three vs. two‐choice response options), which may limit direct cross‐domain comparisons. These domain‐specific effects may be related to extensive deliberate practice of cognitive abilities in the auditory domain involved in music training (i.e., effortful attention, inhibitory control, and auditory working memory) throughout years of instrumental tuning; performance monitoring; correcting patterns of rhythmic, melodic, and harmonic sequences; facilitating transpositions; and aural and written music theory. Complex sensorimotor experiences, such as learning a musical instrument, have been shown to increase preservation of sensorimotor regions of the brain, resulting in enhanced speech‐in‐noise processing (Alain et al. 2014; Zhang et al. 2021, 2023, 2025).

Research in pattern separation with older adults suggests age‐related declines in fidelity of representing highly similar details belonging to separate, yet overlapping items or events at encoding, as primarily demonstrated in tasks of perceptual and mnemonic discrimination in the visual domain (Davidson et al. 2019; Gellersen et al. 2024; Stark et al. 2015; Stark and Stark 2017). Animal models suggest that pattern separation processes are attributed to the dentate gyrus, perirhinal cortex, and lateral perirhinal gyrus (Lim and Lee 2024; Suter et al. 2019). Future work may investigate whether older musicians show preserved structural or functional changes in such hippocampal areas necessary for pattern separation such as the dentate gyrus (Baker et al. 2016), and whether these abilities generalize across sensory domains and explain enhanced inhibitory control in musicians (Amer et al. 2013; Bugos 2010), given reduced interference from competing stimuli (Amer and Davachi 2023).

Performance on the test phase of the AMMT placed demands on pattern separation by testing participants' resistance to interference from similar lure and dissimilar foil sequences, which was shown to increase over time on task. Additionally, older musicians adopted a more conservative response criterion threshold (i.e., less likely to endorse any probe as “old”), suggestive of greater inhibitory control and response suppression when discriminating targets from distractor lures and foils. Interestingly, nonmusicians showed greater variability in foil accuracy than musicians and did not show clear ceiling effects as expected; this finding may be due to large interference effects for the foil probe over time, in addition to less conservative response thresholds. Therefore, musicianship may serve as a protective factor, providing less susceptibility to interference from similar, competing auditory stimuli at both encoding and retrieval. Future work may examine the extent to which enhanced interference control from extensive musical training mediates perceptual and mnemonic discrimination, and whether these effects generalize across auditory and visual domains.

### 4.3 Conclusion

Overall, our findings suggest enhanced perceptual processing in aging adult musicians compared with older nonmusician counterparts. Data suggest that enhancements may be related to the attenuation of sensory deficits that typically occur in neurotypical aging adults. We show that older musicians with long‐term musical engagement may have strengthened internal representations of auditory sensory memory, in addition to more robust mnemonic representations for complex auditory stimuli and reduced susceptibility to interference. As benefits to mnemonic discrimination and old/new recognition were not simply explained by perceptual discrimination ability, findings highlight the role of music training and engagement in supporting cognitive reserve and protecting against age‐related declines in auditory encoding and memory precision.

## Author Contributions

**Jennifer A. Bugos:** conceptualization, data curation, formal analysis, funding acquisition, investigation, writing – original draft, writing – review and editing. **Ricky Chow:** data curation, formal analysis, project administration, visualization, writing – original draft, writing – review and editing. **Shimin Mo:** data curation, investigation, writing – review and editing. **R. Shayna Rosenbaum:** conceptualization, funding acquisition, writing – original draft, writing – review and editing. **Claude Alain:** conceptualization, formal analysis, funding acquisition, writing – original draft, writing – review and editing.

## Funding

This work was supported by the Canada First Research Excellence Fund; Vision: Science to Applications program, York Research Chair (to R.S.R.); Fulbright Scholars Award (to J.A.B); the GRAMMY Museum Grant (to J.A.B. and C.A.); and the Natural Sciences and Engineering Research Council (RGPIN‐2021‐02721 to C.A. and RGPIN‐04238‐2015 to R.S.R).

## Conflicts of Interest

The authors declare no conflicts of interest.

## Supporting information


**Table S1:** Summary of cluster‐based statistics: MST test phase waveforms.

## Data Availability

The data that support the findings of this study will be made publicly available at https://osf.io/tzy9n/.
