# Supplementary material for: Enhanced Contour‐Deviant Mismatch Negativity and Mnemonic Representations in Older Musicians
Source: Eur J Neurosci. 2026 Jun 16;63(12):e70576. doi: 10.1111/ejn.70576 (PMC13272281; doi:10.1111/ejn.70576)
Supplement: Supplementary file 1 — Table S1: Summary of cluster‐based statistics: MST test phase waveforms. [file EJN-63-0-s001.docx]

**Supplementary** **Materials**

**Supplementary Table 1**

*Summary of Cluster-Based Statistics: MST Test Phase Waveforms*

| **Electrode Cluster** | | **Channel at max** | **Max *t*-value** | **Time(ms)** | ***p*-value** |
| --- | --- | --- | --- | --- | --- |
| **Old-New Contrast (Old \| Targets vs. New \| Foils)** | | | | | |
| **Older Musicians** | | | | | |
| **1** | Fp1, AF7, AF3, F1, F3, FC1, Fpz, Fp2, AF8, AF4, AFz, Fz, F2, F4, F6, F8, FT8, FC6, FC4, FC2, FCz, C2, T8, F10, LO1, LO2, IO1, IO2 | AF4 | 7.12 | 355-582 | <.001 |
| **2** | C5, T7, CP5, CP3, CP1, P1, P3, P5, P7, P9, PO7, PO3, O1, Iz, Oz, POz, Pz, CP4, CP2, P2, P4, PO4, O2, PO9, TP9 | O1 | -6.95 | 402-545 | .001 |
| **Older Non-Musicians** | | | | | |
| **1** | F1, FC1, Fp2, AF8, AF4, AFz, Fz, F2, F4, F6, F8, FT8, FC6, FC4, FC2, FCz, C2, C6, T8, CP6, CP4 | FC4 | 5.30 | 432-641 | <.001 |
| **2** | PO7, PO3, Iz, Oz, POz, P4, P6, P8, P10, PO8, PO4, O2, PO10, TP10 | PO8 | -4.78 | 465-529 | .008 |
|  |  |  |  |  |  |
| **False Lure Recognition Contrast (Old \| Lures vs. New \| Foils)** | | | | | |
| **Older Musicians** | | | | | |
| **1** | C3, T7, TP7, CP5, CP3, CP1, P1, P3, P5, P7, P9, PO7, PO3, O1, Iz, Oz, POz, Pz, CPz, C2, C4, C6, T8, TP8, CP6, CP4, CP2, P2, P4, P6, P8, P10, PO8, PO4, O2, PO9, PO10, TP9, TP10 | O2 | -4.77 | 396-846 | <.001 |
| **2** | F1, F3, FC1, C1, FPz, FP2, AF8, AF4, AFz, Fz, F2, F4, F6, F8, FT8, FC6, FC4, FC2, FCz, C2, C6, T8, TP8, P8, FT10, F10, LO2 | F4 | 5.35 | 412-639 | <.001 |
| **3** | FP1, AF7, F1, F3, F5, FT7, FC5, FC1, FPz, FP2, AF8, AFz, F6, F8, FC2, FCz, F9, F10, LO1, IO1 | FC5 | 5.94 | 635-842 | .004 |
| **4** | FP1, AF3, F1, F5, FC5, FC3, FC1, FPz, FP2, AF8, AF4, AFz, F2, F4, F6, F8, FT8, FC6, FC4, FC2, FCz, C2, C4, F10, LO2, IO2 | FC6 | 4.00 | 170-283 | .008 |
| **5** | TP7, P1, P3, P5, P7, PO7, PO3, O1, Oz, POz, Pz, PO4 | O1 | -4.43 | 332-416 | .037 |
| **Older Non-Musicians** | | | | | |
| **1** | F1, AF8, AF4, AFz, Fz, F2, F4, F6, F8, FT8, FC6, FC4, FC2, FCz, C2, C4, C6, T8, CP6 | Fz | 5.39 | 504-658 | <.001 |
| **2** | AF4, AFz, Fz, F2, F4, F6, F8, FT8, FC6, FC4, FC2, FCz, C2, C4, C6, T8 | FC4 | 4.45 | 426-498 | .006 |
| **3** | P7, P9, PO7, PO3, O1, Oz, POz, P4, PO8, PO4, O2, PO9, TP9 | Oz | -4.50 | 428-547 | .009 |
| **4** | P7, P9, PO7, PO3, O1, Oz, POz, PO8, PO4, O2, PO9 | PO7 | -4.38 | 551-598 | .023 |
|  |  |  |  |  |  |

*Note:* Old-New Contrast: Within each group, a pair of clusters identified the frontocentral FN400 and its polarity reversal over parietal-occipital electrodes. False Lure Recognition Contrast: The FN400 was identified within each group. Within the musician group, the FN400 polarity reversal was also identified, as were early visual-evoked responses between conditions due to perceptual differences. Within non-musicians, early and late portions of the FN400 and its polarity reversal were identified. No group difference was identified for the difference waveforms. For ease of interpretation, spurious spatiotemporal clusters (including those exceeding 1000 ms after stimulus onset and overlapping the response time window) are not reported.
